# Temperature–dependent reaction rates of quinone-alkene cycloaddition reveal that only entropy determines the rate of SPOCQ reactions

**DOI:** 10.1039/d5sc04275e

**Published:** 2025-10-29

**Authors:** Johannes A. M. Damen, Jorge Escorihuela, Judith Firet, Han Zuilhof, Bauke Albada

**Affiliations:** a Laboratory of Organic Chemistry, Wageningen University & Research Stippeneng 4 Wageningen 6807 WE the Netherlands bauke.albada@wur.nl han.zuilhof@wur.nl; b Departamento de Química Orgánica, Universitat de València, Facultad de Farmacia y Ciencias de la Alimentación Avda. Vicent Andrés Estellés s/n, Valènciaés Estellés s/n, Burjassot Valéncia 46100 Spain; c China-Australia Institute for Advanced Materials and Manufacturing, Jiaxing University Jiaxing 314001 China

## Abstract

Second-order rate constants and thermodynamic activation parameters for the strain-promoted oxidation-controlled quinone (SPOCQ) click reaction of an *ortho*-quinone with various *trans*-cyclooctene and cyclooctyne reagents were determined by stopped-flow spectroscopic analysis. We substantiate the origin of the enhancements of the reaction rates in various sTCO derivatives as compared to TCO, and demonstrated that *ortho*-quinone-cycloalkene cycloadditions are fully entropy-controlled. The *endo/exo* differences of BCN in SPOCQ and SPAAC were also (re)evaluated, revealing absence of a difference in reactivity between these two isomers for both click reactions. Full crystallographic descriptions of *endo*-BCN-OH and DBCO combined with high-end DFT ring-strain computations confirm that entropy controls this reaction for both cycloalkenes and cycloalkynes alike.

## Abstract 2

Detailed Eyring analysis of the SPOCQ reaction between a quinone and alkene or alkyne dienophiles quantifies the rate constants and reveals that the high reaction rates originate from preorganization of the dienophiles towards the transition state.

## Graphical abstract

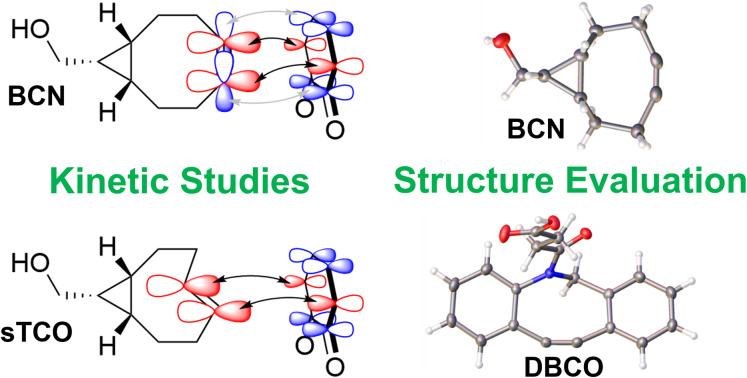

## Introduction

**Fig. 1:**
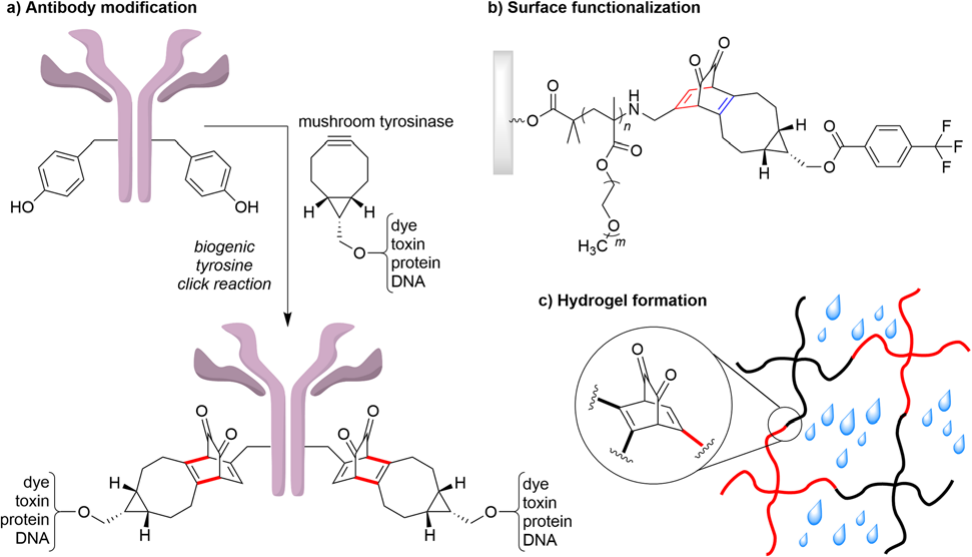
Various applications of the SPOCQ click reaction. (a) in the preparation of antibody conjugates,^5–9^ (b) surface functionalization,^11,12^ and (c) the formation of polyethyleneglycol (PEG)-based hydrogels.^10^

Strain-promoted oxidation-controlled *ortho*-quinone (SPOCQ) alkene/alkyne cycloadditions classify as a [4 + 2] inverse electron-demand Diels–Alder (IEDDA) reaction between the electron-poor diene moiety of a quinone and a strained unsaturated carbon–carbon bond of a cycloalkene or cycloalkyne. Prominent features of this click reaction are reaction rates that are orders of magnitude higher than that of the more commonly applied strain-promoted alkyne–azide cycloaddition (SPAAC), its applicability in various settings, and its inducible nature as the *ortho*-quinone can be generated from a corresponding phenol by oxidation.^1,2^ In the context of protein modification, a tyrosine residue can be oxidized enzymatically, resulting in a ‘biogenic’ click reaction that has been widely used to attach foreign properly functionalized moieties to a protein of interest,^3,4^ such as the conjugation of toxic agents, proteins, and oligonucleic acids to therapeutic antibodies (Fig. 1a).^5–9^ In addition, the SPOCQ reaction has been employed for hydrogel formation and for rapid and quantitative surface modification (Fig. 1b and c).^10–12^ Despite its wide applicability and rapidly increasing use, understanding underlying thermodynamic parameters of various strained cyclic reagents in SPOCQ has been limited to computational studies.^13–16^ Theoretical mechanistic studies postulated the involvement of secondary orbital interactions (SOIs) to rationalize the higher reactivity of strained alkynes (*k*_2_ ∼ 1 × 10^3^ M^−1^ s^−1^)^17^ over that of the strained *trans*-cyclooctene TCO (*k*_2_ ∼ 1 × 10^1^ M^−1^ s^−1^).^15,16^

**Scheme 1:**
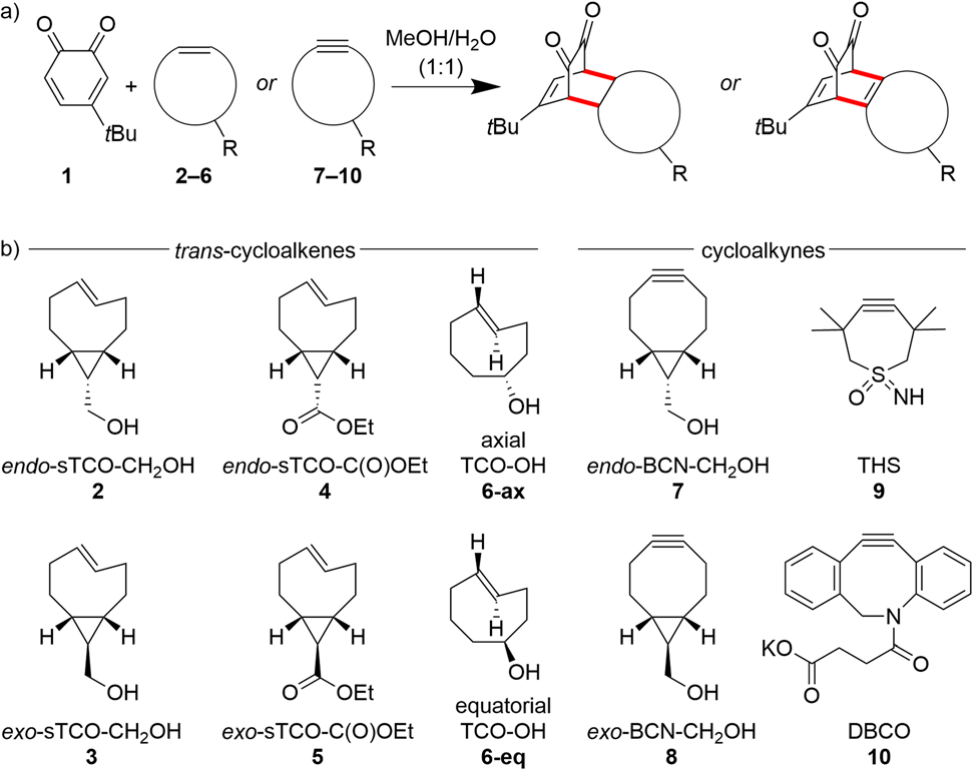
Molecules involved in the inverse electron-demand Diels–Alder reaction between an *o*-quinone diene and strained dienophiles. (a) Generalized scheme for the SPOCQ reaction with diene 1 and dienophiles containing a C=C (2–6) or C≡C (7–10) bond. (b) Matrix of the chemical structures of the cyclic dienophiles 2–10.

Our current paper extensively maps the kinetics and thermodynamic driving forces in SPOCQ click chemistry with a range of cycloalkenes (Scheme 1a), specifically *trans*-cyclooctenol (TCO-OH) and cyclopropanated strained-*trans*-cyclooctene stereoisomers (sTCO; sometimes also referred to as cyclopropanated TCO, cpTCO).^18^ These latter bicyclic derivatives of TCO are structurally similar to BCN while carrying a *trans*-alkene double bond instead of the alkyne triple bond, allowing direct comparison of their reactivities and thermodynamic activation parameters. Temperature-dependent stopped-flow kinetics were used to acquire the second-order rate constants (*k*_2_) whereas thermodynamic activation parameters Δ*H*^‡^ and Δ*S*^‡^ were obtained from Eyring analyses. With this extended matrix (Scheme 1b), the following structural features were addressed: (i) effects of chirality of exocyclic tethering points (*exo versus endo*); (ii) effect of axial or equatorial position of alcohol in TCO-OH; (iii) effects of different functional groups (alcohol *versus* ester) opposite to the dienophile; and (iv) effect of the annulation of *cis*-cyclopropane rings onto the cyclic backbone.^18,19^

## Results and discussion

### Kinetic studies of the SPOCQ reaction

**Fig. 2:**
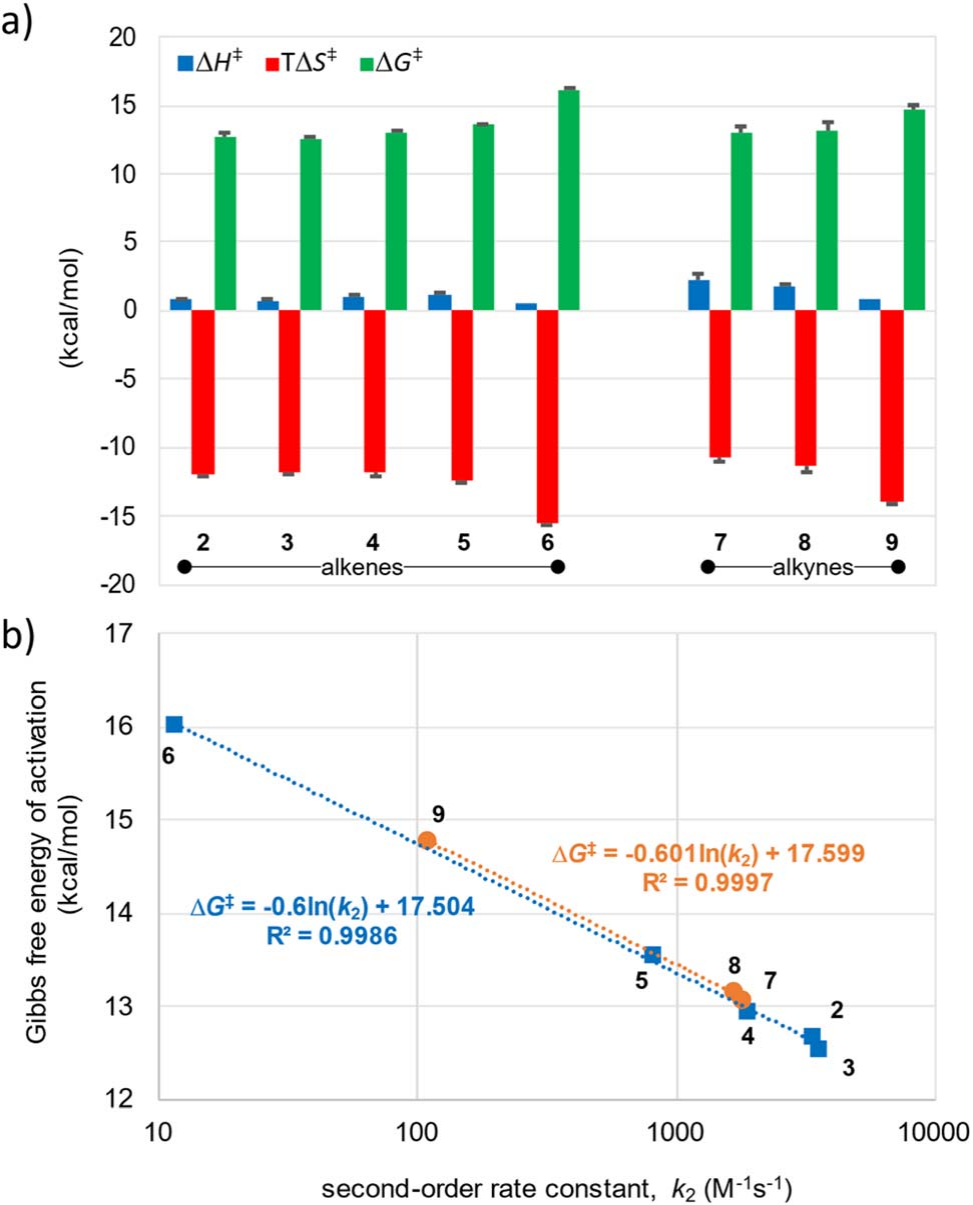
(a) Thermodynamic activation parameters determined from the Eyring plots for the SPOCQ reaction between 4-*tert*-butyl-1,2-quinone (1) and strained alkenes (2–6) (left) and alkynes (7–9) (right). (b) High correlation of the independently determined Δ*G*^‡^ and second-order rate constant for the SPOCQ reaction of the strained cycloalkenes (blue) and cycloalkynes (orange) shows accuracy of our stopped-flow method over the range of reaction rates.

The second-order rate constants for the SPOCQ reaction between 4-*tert*-butyl-1,2-*ortho*-quinone 1 and dienophiles 2–10 (Scheme 1b) were determined under pseudo-first order conditions by means of stopped-flow UV-Vis spectroscopy in a 1 : 1 (v/v) MeOH/H_2_O mixtures at 25 °C. Eyring analyses were performed under similar conditions while maintaining steady temperatures at 8-centigrade intervals in the range between 5–37 °C. The obtained kinetic and thermodynamic data are provided in Table 1 and Fig. 2 (see also SI, Appendix A–D). Our Δ*G*^‡^ barriers are similar to experimentally determined values for other IEDDA click reactions, *e.g.*, those between sTCO and tetrazine.^18^ The large negative Δ*S*^‡^ values in all cases suggests that the SPOCQ cycloaddition is an associative reaction. Whereas all reactions display extremely small enthalpies of activation (Δ*H*^‡^ ranges from 0.7–2.3 kcal mol^−1^), the entropies of activation are not only larger but also vary substantially over the dataset (*T*Δ*S*^‡^ ranges between −10.8 and −15.5 kcal mol^−1^, see Table 1).

Eyring analysis of the SPOCQ reaction of quinone 1 with the racemic mixture of TCO-OH 6 (*k*_2_ = 1.2 × 10^1^ M^−1^ s^−1^) shows that the energy barrier consist of an extremely small enthalpic contribution (Δ*H*^‡^ = 0.5 (±0.1) kcal mol^−1^) and a more substantial entropic contribution (Δ*S*^‡^ = −51.9 (±0.4) cal K^−1^ mol^−1^; *T*Δ*S*^‡^ = −15.5 kcal mol^−1^ at 25 °C), resulting in an overall Gibbs free energy of activation (Δ*G*^‡^) of 16.0 kcal mol^−1^ at 25 °C. Analysis of the two isolated diastereomers, *i.e.*, TCO-OH_ax_ (6-ax) and TCO-OH_eq_ (6-eq), revealed that the former was 12.5-times more reactive in SPOCQ with quinone 1 than the latter (*k*_2,__6-eq_ = 2.8 *vs. k*_2,__6-ax_ = 34.8 M^−1^ s^−1^), which is in line with earlier higher rates for the axial diastereomer.^20^ Whereas Eyring analysis of the equatorial diastereomer was hampered by its slow rate, this analysis for the axial diastereomer revealed the following parameters: Δ*H*^‡^ = 2.1 kcal mol^−1^, Δ*S*^‡^ = −44.4 cal K^−1^ mol^−1^; *T*Δ*S*^‡^ = −13.2 kcal mol^−1^ (at 25 °C) and an overall Gibbs free energy of activation (Δ*G*^‡^) of 15.3 kcal mol^−1^ at 25 °C.

Moving from TCO to sTCO revealed that installation of an annulated *cis*-cyclopropane ring opposite to the dienophile in *trans*-cyclooctene enhances its SPOCQ reactivity >300 fold, resulting in *exo*-sTCO-CH_2_OH 3 as the most reactive reagent in the entire set. This compound displays a *k*_2_ value of 3.5 × 10^3^ M^−1^ s^−1^ and thus reacts twice as fast as its *exo*-BCN-CH_2_OH 8 counterpart; it is one order of magnitude slower than its reaction with a diphenyltetrazine (*k*_2_ = 3.3 × 10^4^ M^−1^ s^−1^).^21^ Interestingly, the enthalpies of activation are near-identical for TCO-OH 6 and *exo*-sTCO-CH_2_OH 3 (ΔΔ*H*^‡^ = 0.1 kcal mol^−1^, which is within the standard deviation of both values, see Table 1).

**Table 1:** Thermodynamic activation parameters and second-order rate constants for the inverse electron-demand Diels–Alder SPOCQ cycloaddition between 4-*tert*-butyl-1,2-*ortho*-quinone 1 and different strained cycloalkenes (2–6) or cycloalkynes (7–9) as determined by Eyring analysis in a water–MeOH mixture (1 : 1, v/v)

Dienophile	Eyring plot				*k*_2_ plot^b^
	Δ*H*^‡^ (kcal mol^−1^)	Δ*S*^‡^ (cal K^−1^ mol^−1^)	*T*Δ*S*^‡,^^b^ (kcal mol^−1^)	Δ*G*^‡,^^b^ (kcal mol^−1^)	*k*_2_ (M^−1^s^−1^)
**Cycloalkenes**					
*endo*-sTCO-CH_2_OH (2)	0.8 (±0.2)	−40.0 (±0.7)	−11.9	12.7	(33.5 ± 0.5)·10^2^
*exo*-sTCO-CH_2_OH (3)	0.7 (±0.1)	−39.8 (±0.4)	−11.9	12.5	(35.3 ± 0.5)·10^2^
*endo*-sTCO-C(O)OEt (4)	1.1 (±0.2)	−39.9 (±0.6)	−11.9	13.0	(18.7 ± 0.8)·10^2^
*exo*-sTCO-C(O)OEt (5)	1.1 (±0.1)	−41.7 (±0.2)	−12.4	13.6	(8.2 ± 0.2)·10^2^
TCO-OH (6)	0.5 (±0.1)	−51.9 (±0.4)	−15.5	16.0	(11.6 ± 0.1)^a^
TCO-OH axial (6-ax)	2.1 (±0.2)	−44.4 (±0.3)	−13.2	15.3	(34.8 ± 0.8)

**Cycloalkynes**					
*endo*-BCN-CH_2_OH (7)	2.3 (±0.3)^a^	−36.3 (±0.9)^a^	−10.8^a^	13.1^a^	(18.2 ± 0.2)·10^2^^a^
*exo*-BCN-CH_2_OH (8)	1.7 (±0.4)	−38.3 (±1.5)	−11.4	13.2	(16.8 ± 0.1)·10^2^
THS (9)	0.8 (±0.2)^a^	−46.9 (±0.6)^a^	−14.0^a^	14.8^a^	(11.1 ± 0.2)·10^1^^a^

a These values were reported previously.^17^

b Determined at 25 °C.

Our data shows that the difference in the entropic component of the barrier accounts for the difference in free energy of activation. Specifically, the value for Δ(*T*Δ*S*^‡^) for TCO-OH 6 is 3.6 kcal mol^−1^ larger than that for *exo*-sTCO-CH_2_OH 3, resulting in ΔΔ*G*^‡^ = −3.4 kcal mol^−1^. From this we conclude that the reactant complex of *o*-quinone 1 with *exo*-sTCO-CH_2_OH 3 is already more preorganized towards the transition state (TS) when compared to TCO-OH 6. A similar analysis can be made when TCO-OH 6 is compared to *endo*-sTCO-CH_2_OH 2. As such, our results support the hypothesis by Fox that the *cis*-fused cyclopropane on sTCO results in a “half-chair”-conformation that is higher in enthalpy than the typical TCO crown conformation,^18^ and better preorganized towards the TS, resulting in a lower entropic barrier. For all our tested sTCO derivatives a substantially lower entropic barrier was found than for TCO-OH 6 (difference ranging from 3.1–3.6 kcal mol^−1^), whilst maintaining only minimal enthalpic barriers (ranging from 0.7–1.1 kcal mol^−1^). Apparently, preorganization towards the TS enhances the rates of these dienophiles in SPOCQ.

We also measured the SPOCQ reaction to DBCO 10, derivatives of which are frequently used in SPAAC-based reactions while sparingly being applied in SPOCQ-based bioconjugation.^22–24^ For this, pseudo-first order conditions with a 10-fold excess of DBCO-acid 10 (as potassium salt) were applied, resulting in a *k*_2_ value of 1.7 × 10^−1^ M^−1^ s^−1^ (corresponding to a calculated Gibbs free energy of activation of approximately 18.6 kcal mol^−1^, based on the equation in Fig. 2b), which is four orders of magnitude less reactive than *endo*-BCN-CH_2_OH 7. Eyring analysis was not possible due to the substantially slower rate and associated requirement for stringent long-term temperature control.

To address the effect of the substituents opposite to the dienophile, the ester analogues of sTCO, *endo*-sTCO-C(O)OEt 4 and *exo*-sTCO-C(O)OEt 5 were measured. This revealed similar high reactivity in SPOCQ reactions, albeit lower than their alcohol counterparts. Interestingly, we observed a two-fold difference in reactivity between *endo*-sTCO-C(O)OEt 4 (1.9 × 10^3^ M^−1^ s^−1^) and *exo*-sTCO-C(O)OEt 5 (0.8 × 10^3^ M^−1^ s^−1^), even in repeated experiments using new batches of these dienophiles. For *exo*-sTCO-C(O)OEt 5 an additional increase in entropy of activation is found, while the enthalpy of activation is the same as for the *endo* counterpart (Δ*H*^‡^ = 1.1 kcal mol^−1^). This slightly higher entropic barrier (Δ(*T*Δ*S*^‡^) = 0.5 kcal mol^−1^) is reflected in the overall reaction barrier (ΔΔ*G*^‡^ = 0.6 kcal mol^−1^), resulting in the observed *ca.* 2.3-fold difference in reactivity.^25^ The difference in activation entropy between the *endo* and *exo* isomer is tentatively attributed to interaction of the C(O)OEt group with the alkene, although the exact origin is unknown.

The effect of solvent on the kinetics and associated thermodynamic activation parameters was analyzed on a subset of dienophiles in MeOH. First, the second-order rate constant for the most hydrophobic dienophiles, *i.e.*, *endo*-sTCO-C(O)OEt 4 and *exo*-sTCO-C(O)OEt 5 (25 °C), revealed that *k*_2_ values were an order of magnitude lower than in the water–MeOH mixture (*k*_2,(__4__)_ = 9.5 *vs. k*_2,(__5__)_ = 39.6 M^−1^ s^−1^). Interestingly, the second-order rate constants for *endo*-sTCO-CH_2_OH 2 and *exo*-sTCO-CH_2_OH 3 in MeOH were in a similar range, with *k*_2,(2)_ = 58.0 M^−1^ s^−1^ and *k*_2,(3)_ = 32.9 M^−1^ s^−1^, but also now showing a notable difference between *endo* and *exo*. Eyring analysis of sTCO 2 and 3 revealed a slightly higher but still small enthalpic contribution for both (Δ*H*^‡^ = 1.1 kcal mol^−1^) and a more pronounced differences in the larger entropic contribution (Δ*S*^‡^ = −46.8 cal K^−1^ mol^−1^ for *endo*-sTCO-CH_2_OH 2 and Δ*S*^‡^ = −47.8 cal K^−1^ mol^−1^ for *exo*-sTCO-CH_2_OH 3; which corresponds to *T*Δ*S*^‡^ values at 25 °C of −14.0 kcal mol^−1^ for *endo*-sTCO-CH_2_OH 2 and −14.3 kcal mol^−1^ for *exo*-sTCO-CH_2_OH 3).^26^ This result in an overall Gibbs free energy of activation (Δ*G*^‡^) of 15.1 kcal mol^−1^ for *endo*-sTCO-CH_2_OH 2 and of 15.4 kcal mol^−1^ for *exo*-sTCO-CH_2_OH 3 (at 25 °C). The increased entropic values point to a more ordered transition state in MeOH when compared to the water–MeOH mixture (1 : 1, v/v).^26^

### Revisiting the influence of BCN isomer in SPAAC

**Scheme 2:**
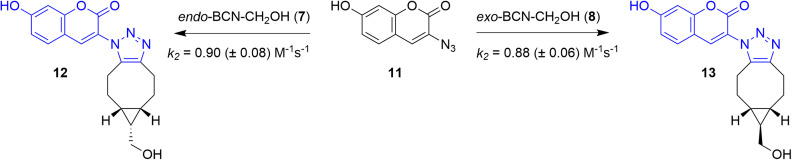
Reaction of *endo*-BCN-CH_2_OH 7 and *exo*-BCN-CH_2_OH 8 with 3-azido-7-hydroxycoumarin 11 to form the fluorescent SPAAC products 12 and 13, respectively, proceeds with identical second-order rate constants.

As we observed that the *exo* and *endo* isomers of sTCO-CH_2_OH did not display a significant difference in their reactivity towards *o*-quinone 1 nor in the underlying activation parameters between its isomers (*k*_rel,*exo*/*endo*_ = 1.1), which was previously also observed for their reactivity towards tetrazines,^21^ we evaluated the kinetics of the *exo* and *endo* isomers of the eight-membered cycloalkyne BCN. First, we determined the SPOCQ kinetics of *exo*-BCN-CH_2_OH 8 with diene 1, and compare this with our previously reported values for *endo*-BCN-CH_2_OH 7.^17^ We found that *exo*-BCN-CH_2_OH 8 exhibits a second-order rate constant *k*_2_ of 1.7 × 10^3^ M^−1^ s^−1^, which is near-identical to what we determined for *endo*-BCN-CH_2_OH 7 (*k*_2_ of 1.8 × 10^3^ M^−1^ s^−1^). Also, we did not detect a notable difference in the thermodynamic activation parameters associated to both isomers. This is in sharp contrast to an earlier claim regarding a notable difference in reactivity between the *exo*/*endo* isomers of BCN–CH_2_OH in SPAAC reactions.^19^ Therefore, we revisited this claim by determining the reactivity of both BCN isomers with an organic azide in our stopped-flow UV-Vis equipment. As overlap of the absorption bands of both the azide and the formed triazole product at 213 nm prevented UV-Vis measurements of this transformation, we used the known fluorogenic substrate 3-azido-7-hydroxycoumarin 11. Specifically, azide 11 was reacted with *endo*-BCN-OH 7 or *exo*-BCN-CH_2_OH 8 under pseudo-first order conditions in MeOH/H_2_O (1 : 1) to form the fluorescent triazole product (Scheme 2).^27^ We found that *endo*-BCN-CH_2_OH 7 reacted with a *k*_2_ value of 0.90 (±0.08) M^−1^ s^−1^ and *exo*-BCN-CH_2_OH 8 with a *k*_2_ value of 0.88 (±0.06) M^−1^ s^−1^, resulting in a *k*_*rel*,*endo*/*exo*_ of 1.02 (see SI, Appendix E). Therefore, we were not able to confirm the reported difference between *exo* and *endo*-BCN-CH_2_OH in SPAAC click chemistry.^28^ Together with our analogous observation on the SPOCQ reaction, we conclude that it is likely that the reported difference in reactivity of *endo*-BCN and *exo*-BCN did not originate from intrinsic differences in reactivities of the two isomers of BCN. We conclude that the stereochemistry of the annulation is an ineffective approach to influence the reaction rate of SPAAC and SPOCQ reactions.

### Crystal structure of *endo*-BCN-CH_2_OH (7) and DBCO (10)

**Fig. 3:**
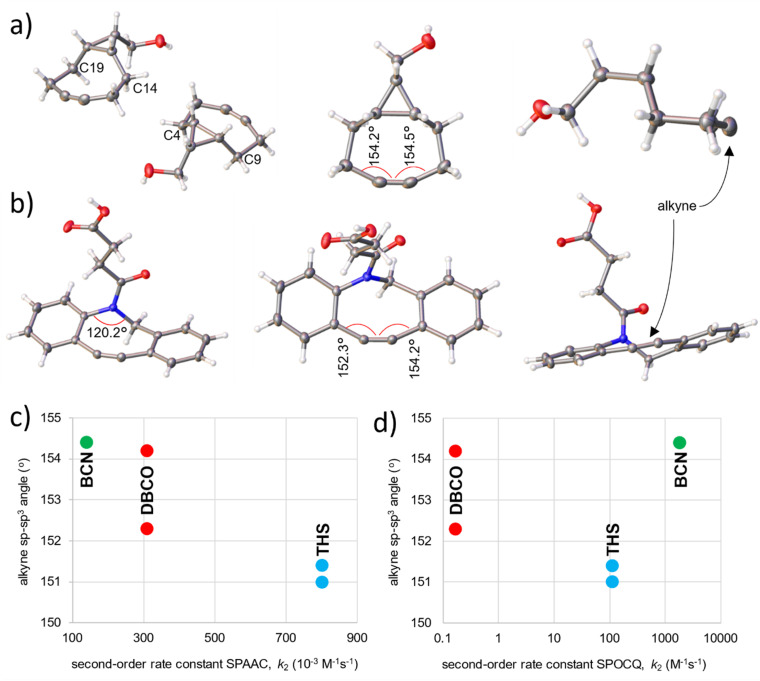
(a) The molecular structure of *endo*-BCN-CH_2_OH 7 according to X-ray structure determination. ORTEP depicted with thermal ellipsoids drawn at 50% probability level. The panel shows the presence of two molecules in the unit cell (left), and top and side view of isolated BCN molecules (*middle* and right); atoms and bond angles that are mentioned in the text are indicated. (b) The molecular structure of DBCO-acid 10 according to X-ray structure determination. ORTEP depicted with thermal ellipsoids drawn at 50% probability level. The panel shows DBCO from the side, top and front (left-to-right); bond angles that are mentioned in the text are indicated. (c) Chart showing a correlation between the strained alkyne bond angles, and the second-order rate constant for the SPAAC reaction (*R*^2^ = 0.9875). (d) Chart showing the absence of a correlation between the strained alkyne bond angles, and the second-order rate constant for the SPOCQ reaction (*R*^2^ = 0.5470). The ORTEP figures were generated with Olex2.^34^

Structural details for the BCN and DBCO skeletons were obtained by X-ray diffraction on single crystals. Suitable crystals of *endo*-BCN-OH (7) were grown from a concentrated hot Et_2_O solution, for DBCO (10) this was from a hot THF solution. The resulting crystal structures are depicted in Fig. 3a and b; unfortunately, we were not able to grow crystals from *exo*-BCN-CH_2_OH (8).

Regarding the structure of *endo*-BCN-CH_2_OH (7) (Fig. 3a), the endocyclic cyclopropane bond length is 1.52 Å, which is typical for sp^3^–sp^3^ hybridized carbon atoms. The structure reveals that ring pinching is afforded by means of reducing the interatomic distance between the two methylene groups that are attached to the cyclopropane ring and those next to the alkyne bond, *i.e.*, C14–C19 and C4–C9 to 3.277 Å and 3.294 Å, respectively (see SI, Appendix F). These interconnecting methylene groups of the propargylic position bend inwards to 105.2°–105.5° and of those next to the cyclopropane ring outwards to 112.0°–112.8°, which deviate substantially from 109.3° bond angles expected for sp^3^ carbon atoms,^29^ forming a strained plane within the molecule. This forces BCN into a conformation in which the alkyne is oriented in a coplanar fashion (only 1.2° torsional angles, see Fig. 3a, structure on the right), resulting in bond angles of 154.2°–154.5° between the sp and sp^3^ hybridized carbon atoms. These angles are markedly narrower than the value of 158.5° that was reported for the cyclooctyne parent compound.^30^ Therefore, the enhanced reactivity of BCN over that of cyclooctyne is caused by an increase in angular strain of the alkyne.^19,31^

Inspection of the crystal structure of DBCO-acid 10 (Fig. 3b) reveals that the two individually planar aromatic rings are tethered together by a heavily distorted alkyne functionality, which is unsymmetrically bent at 152.3° and 154.2° (see SI, Appendix G). The alkyne possesses dihedral distortion by means of a 17.0° torsional angle, resulting in an antiparallel positioning of the aromatic planes relative to each other. The other bridge between the two rings is formed by a nitrogen atom and a methylene group, displaying a dihedral angle of 121.6°. Whereas the methylene group has a bond angle of 115.6°, thereby deviating from normal sp^3^ geometry, the nitrogen atom has an endocyclic bond angle of 120.2°, confirming sp^2^ hybridization of this amide (sum of all bond angles around the endocyclic nitrogen atom is 359.6°). Interestingly, the attachment to the amide protrudes in an axial fashion from the DBCO ring system.

### Alkyne bond angle and reactivity correlation

The XRD structures of *endo*-BCN-CH_2_OH (7) and DBCO (10) (Fig. 3a and b) and of the earlier published structure of THS (9)^17^ allow assessment of the importance of bond angles of the involved disubstituted alkynes in SPAAC and SPOCQ click reaction kinetics. THS possesses symmetrical alkyne bond angles of 151.0°–151.4° and is therefore the most strained stable and structurally well-characterized cycloalkyne to date.^17^ As described before, those of *endo*-BCN-CH_2_OH (7) are 154.2°–154.5° and those of DBCO (10) are 152.3° and 154.2°. Plotting these angles to reported second-order rate constants for [3 + 2] SPAAC reveals a correlation with *R*^2^ = 0.9875 (Fig. 3c).^32^ Such nice correlation is not found for the [4 + 2] SPOCQ reactions, where this plot shows *R*^2^ = 0.5470 (Fig. 3d). It appears that the planar TS of [3 + 2] SPAAC –in which linear azide and alkyne form a planar triazole– fundamentally differs from the three-dimensionally demanding TS of [4 + 2] SPOCQ reactions as the flat ring of the quinone is converted into an out-of-plane bent structure of the resulting bicyclo[2,2,2]octadiene. Our data shows that the height of the TS barrier in SPOCQ is primarily dictated by entropy and that reagent bulkiness has to be taken into account. For example, we previously ascribed the lower reactivity of THS with respect to BCN in SPOCQ to the presence of substantial steric bulk originating from the methyl groups neighboring the alkyne functionality.^17^ A similar reasoning can be applied for DBCO (10), which is even less reactive, as the presence of the aromatic rings that flank the alkyne in DBCO hinder its interaction with the quinone diene, suppressing the reaction rate constant.

Following Hammond's Postulate, structures of TSs often resemble the molecular structures of the reagents in the case of an early TS exergonic reaction. This means that the net bond breaking plus bond formation energy barrier (Δ*H*^‡^) would be lower for more strained systems, which is also seen in our dataset, *e.g.*, by comparing THS and *endo*-BCN-CH_2_OH (ΔΔ*H*^‡^ = 1.4 kcal mol^−1^).

**Fig. 4:**
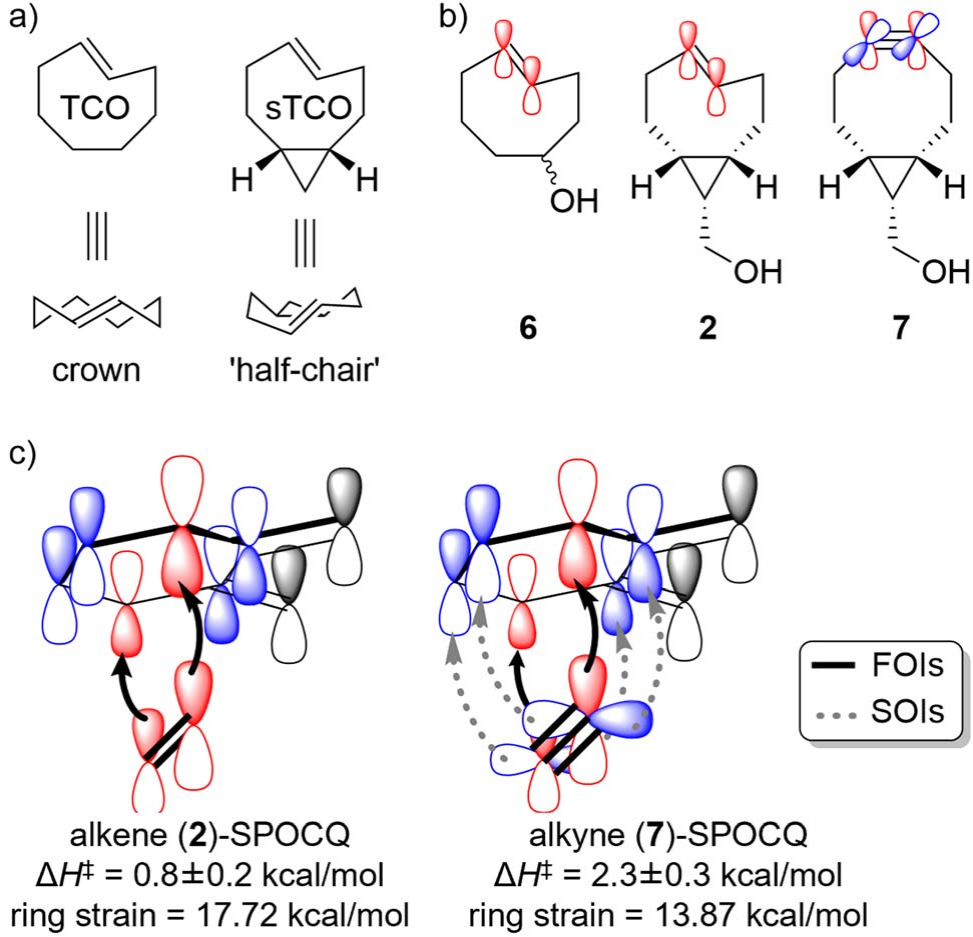
Structural considerations regarding the interpretation of the thermodynamic activation parameters. (a) Effect of annulated cyclopropane ring on the conformation of *trans*-cyclooctene. (b) Graphical representation of the p-orbitals of the C=C bonds in TCO-OH (6) and *endo*-sTCO-CH_2_OH (2) and of the C≡C bond in *endo*-BCN-CH_2_OH (7). (c) Schematic depiction of the frontier orbital interactions (FOIs) of the alkene-SPOCQ and alkyne-SPOCQ reactions (between red p-orbitals, indicated by black arrows), and of proposed secondary orbital interactions (SOIs) of the alkyne-SPOCQ reaction (between the blue orbitals, indicated by grey arrows). Oxygen atoms in the diene and the ring of the dienophile are omitted for clarity.

A similar, but distinct phenomenon is observed for *trans*-cyclooctenes. The crown-like conformation of the molecule enforces dihedral bending of the sp^2^–sp^2^ bond plane of the alkene to an extent of 133.0°, as was shown crystallographically for an *O*-alkylated version of TCO-OH (6) by Fox *et al.* (Fig. 4a).^33^ This 47° geometrical distortion of alkene planarity likely contributes strongly to the extremely low Δ*H*^‡^ values found for cycloalkene SPOCQ reactions (Table 1). Annulation of the cyclopropane ring to TCO raises the energy of the structure by forcing it into a “half-chair”-conformation (Fig. 4a). In the absence of crystal structure information, the effect of this on the geometrical distortion of the alkene planarity is not known. However, as the activation enthalpy of both sTCO-CH_2_OH structures, *i.e.*, 2 and 3, is statistically indifferent from that of TCO, it can be assumed that a similar distortion of the double bond is also present in the sTCO family of dienophiles. Thus, the presence of the cyclopropane ring likely does not distorts the alkene more than is already the case in TCO, but has a larger effect of the conformation of the ring structure, as is also apparent from the large differences in the entropies of activation between TCO and the sTCO family, *i.e.*, −15.5 kcal mol^−1^ for TCO-OH (6) *vs.* −11.9 kcal mol^−1^ for both *endo*-sTCO-CH_2_OH (2) and *exo*-sTCO-CH_2_OH (3).

Lastly, we observed that the Δ*H*^‡^ for sTCO-CH_2_OH are slightly but significantly lower than those for the BCN–CH_2_OH counterpart, with 0.6–1.0 kcal mol^−1^*versus* 1.3–2.5 kcal mol^−1^ (standard deviations are included in these values), respectively. As such, involvement of secondary orbital interactions (SOIs) are not reflected in the enthalpy of activation in SPOCQ (Fig. 4b and c).

## Computational investigations

The rather low activation enthalpies –which were also confirmed by computational analysis (see SI, Table S20)– also suggest that our dienophiles do not show a direct link between reaction rate and ring strain. To verify this, high-end calculations of the ring strain in the dienophiles (at the wB97M-V/def2-QZVPP//M06-2X/6-311+G(d,p) level of theory) were performed by calculating the free energy of ring-opened analogues of the ring structures under current study and those of the cycloalkenes and cycloalkynes for which we have activation parameters. These values were then compared to those of *n*-hexane and cyclohexane, as ring-shaped and ring-opened analogs that both did not display ring strain (see SI for details). These calculations show that ring strain is higher for the more reactive *trans*-cyclooctenes than for the less reactive cyclooctynes, and that this parameter varies gradually over the range of compounds within a range of *ca.* 5 kcal mol^−1^ (Table 2). However, there is no correlation between the ring strain and the activation enthalpy or activation free energy, and thus not with the reaction rate. In other words: in all these systems there is already sufficient ring strain to pre-load the reactants with such a drive that not enthalpy but entropy determines the reactions rates. Interestingly, the 7-membered THS 9 has, despite its smaller C–C≡C bond angles, an intermediate value for its ring strain, and not reaching those of our sTCO derivatives, which are clearly more strained than cyclooctynes like BCN. Comparison of the calculated ring strain with heats of hydrogenation calculated by the same method indeed reveals that THS 9 is in reactivity more similar to the cycloalkenes 2–6 than to the cycloalkynes BCN–CH_2_OH 7 and 8 (see SI, Fig. S3).

**Table 2:** Ring strain calculations of different strained dienophiles 2–9 at the wB97M-V/def2-QZVPP//M06-2X/6-311+G(d.p) level of theory

Dienophile	Ring strain (kcal mol^−1^)
**Cycloalkenes**	
*endo*-sTCO-CH_2_OH 2	17.72
*exo*-sTCO-CH_2_OH 3	17.25
*endo*-sTCO-C(O)OEt 4	18.26
*exo*-sTCO-C(O)OEt 5	17.60
TCO-OH 6	18.41

**Cycloalkynes**	
*endo*-BCN-CH_2_OH 7	13.87
*exo*-BCN-CH_2_OH 8	14.53
THS 9	16.73

## Conclusions

Temperature-dependent kinetic studies using stopped-flow UV-Vis spectroscopy provided detailed insights into *o*-quinone–cycloalkene click chemistry. We found that the Δ*H*^‡^ values amongst the TCO dienophiles are all extremely small (<2 kcal mol^−1^) with minimal differences within the series, and that mainly the activation entropy Δ*S*^‡^ dictates the height of the Δ*G*^‡^ barrier. Introduction of a cyclopropane moiety opposite to the dienophile in the eight-membered ring enforces reactivity and reduces the entropic barrier. We substantiated the reactivity of axial-TCO-OH and equatorial-TCO-OH with measured *k*_2_ values of 34.8 M^−1^ s^−1^ and 2.8 M^−1^ s^−1^, respectively, a 12.5-fold difference in reactivity in SPOCQ with quinone 1 in favour of the axial diastereomer. This settles the sTCO class of dienophiles as the new benchmark reagents in SPOCQ chemistry, reaching *k*_2_ values of 3.5 × 10^3^ M^−1^ s^−1^ for the alcohol-functionalized version 2 and 3. Our reaction kinetics studies in MeOH reveal a strong on water-effect on the kinetics, as the second-order rate constants are approximately two orders of magnitude lower than in water–MeOH, 1 : 1 (v/v). Eyring analysis in MeOH revealed a similar value for the enthalpy of activation, but a substantially larger entropic barrier when compared to the water–MeOH mixture. Furthermore, no reactivity differences were found between *exo* and *endo* diastereomers of the probes in SPOCQ, unless bulkier substituents were positioned opposite of the dienophile. The long-claimed difference between *endo*-BCN-CH_2_OH (7) and *exo*-BCN-CH_2_OH (8) in SPAAC chemistry was also found not to be true. Furthermore, XRD analysis of both BCN and DBCO allowed us to conclude that the higher angular tension of DBCO does not affect its reactivity in SPOCQ, as BCN is 11 000 times more reactive, but that the bond angles of the disubstituted alkynes do correlate to the reaction rates observed for SPAAC. Finally, high-level DFT calculations yield as order of ring strains: sTCOs (17–18 kcal mol^−1^) > cycloheptyne THS (16 kcal mol^−1^) > BCN derivatives (14–15 kcal mol^−1^). The absent correlation between ring strain and Δ*H*^‡^ or Δ*G*^‡^ confirms that the ring strain in these compounds is already sufficient to reduce the Δ*H*^‡^ to insignificantly small values, and that ‘reducing chaos’ determines the reaction rate in SPOCQ chemistry. By uncovering the thermodynamic activation parameters of this SPOCQ click reaction, determining the crystal structure of the compounds, and calculations of the ring strain, we were able to construct a theoretical framework that enable future developments to push this unique set of biogenic click reactions into untapped domains, especially when high reaction rates are required.^2^

## Methods

### Stopped-flow UV-Vis kinetic studies

The reaction of 4-*tert*-butyl-*ortho*-quinone 1 with dienophiles 2–10 was measured under pseudo-first order conditions in 1 : 1 MeOH/MilliQ water following the decay of the specific absorption band at 395 nm for *o*-quinone 1. Two respective equivolume solutions of *o*-quinone 1 and the probe of interest, in MeOH : water −1 : 1 (v/v) or in pure MeOH, were loaded into the two separate driver syringes of the RX2000 Rapid Kinetics Spectrometer Accessory (Applied Photophysics). The accessory is attached to a thermostat bath and to a Cary 60 UV-Vis spectrophotometer. The solutions in the driver syringes were thermostatted for at least 15 min prior to measurement. Upon measurement, the contents of the two driver syringes were flown simultaneously though the cuvette and measurement starts upon abruptly stopping the flow. Single wavelength measurements were then recorded every 12.5 ms at 395 nm. The measurements were performed in quadruplicate until the signal stabilizes. This setup utilizes equal volumes of the reagents, thereby halving each respective concentration in the cuvette. Concentrations are hereby referred to as final concentrations in the reaction mixture. The experiments were conducted using 40 μM solutions of *o*-quinone 1 (1 eq) and 0.4–4 mM solutions of dienophile 2–9 (*i.e.*, 10–100 eq.) to allow for acquisition of sufficient data points for analysis. From these, *k*_2_ plots were determined at 25 °C with the varying stoichiometry of the target probes. Eyring plots were determined at a set stoichiometry of 1 : 10 at varying temperatures of 5, 13, 21, 29, 37 °C. Measurements for DBCO-acid 10 (as potassium salt) were performed at a higher concentration of 4 mM DBCO with 0.4 mM *o*-quinone 1 at a set stoichiometry of 1 : 10 equivalents. Data analysis was then performed in GraphPad Prism 9 Version 9.3.1 (471) by exponential one phase decay fitting using nonlinear regression until a plateau of constant value is reached, leading to an observed pseudo-first-order rate constant *k*’ (see SI for additional details). The *k*_2_ values were then determined from the slope of the linear *k*’ *versus* [dienophile] plot. The thermodynamic activation parameters Δ*H*^‡^ and Δ*S*^‡^ were determined *via* the classic method of Eyring utilizing the following linearized equation, with transmission coefficient *κ* (equals one); Boltzmann constant *k*_B_; Planck's constant *h*; gas constant *R*; temperature *T* (in K).^35–39^
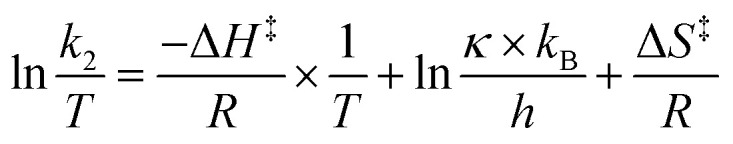


### Fluorescence spectroscopy kinetic studies

Fluorescence measurements were performed on a Edinburgh Instruments FLS900 Fluorescence spectrometer at 20 °C under pseudo-first order conditions in 1 : 1 MeOH/MilliQ water (v/v). Two equivolume solutions of 10 μM 3-azido-7-hydroxycoumarin 11 and 10 mM *endo*-BCN-CH_2_OH 7 or *exo*-BCN-CH_2_OH 8 were mixed in a quartz cuvette at a set stoichiometry of 1 : 1000 equivalents. Concentrations are referred to as final concentrations as in the reaction mixture. Formation of the fluorescent triazole click product was followed over time (*λ*_ex_ = 395 nm; *λ*_em_ = 472 nm). Emission spectra were recorded every 9 seconds for 15 minutes at 472 nm, at which point the increase of signal reached a plateau. Data analysis was then performed in GraphPad Prism 9 Version 9.3.1 (471) by exponential plateau fitting using nonlinear regression, leading to an observed pseudo-first-order rate constant *k*’, from which the *k*_2_ values were then obtained by dividing *k*’ by [*endo*-BCN-CH_2_OH] or [*exo*-BCN-CH_2_OH]. The measurements were performed in triplicate for each compound.

## Abbreviations

ax: Axial
BCN: Bicyclo[6.1.0]non-4-yne
DBCO: Aza-dibenzocyclooctyne
eq: Equatorial
Δ*G*^‡^: Gibbs energy of activation
Δ*H*^‡^: Enthalpy of activation
Δ*S*^‡^: Entropy of activation
*k*_2_
: Second-order rate constant
SOI: Secondary orbital interaction
SPAAC: Strain-promoted (3 + 2) azide–alkyne cycloaddition
SPOCQ: Strain-promoted oxidation-controlled *ortho*-quinone cycloaddition
sTCO: Strained-*trans*-cyclooctene
TCO: *trans*-Cyclooctenol
THS: 3,3,6,6-Tetramethyl-1-thiacyclo-heptyne sulfoximide
TS: Transition state

## Author contributions

J. A. M. D. performed most experiments in water–MeOH and grew the single crystals for X-ray analysis, J. F. performed all experiments in MeOH, J. E. and H. Z. performed the computational analysis, B. A. conceived the project, J. A. M. D. and B. A. wrote the manuscript, all authors delivered input for the manuscript and approved the final version.

## Conflicts of interest

The authors declare no competing financial interest.

## Supplementary Material

SC-016-D5SC04275E-s001

SC-016-D5SC04275E-s002

## Data Availability

Crystallographic data for structures of compounds 7 and 10 have been deposited at the Cambridge Crystallographic Data Centre (CDCC) under deposition numbers 2378937 for *endo*-BCN-CH_2_OH (7) and 2378938 for DBCO (10)). CCDC 2378937 and 2378938 contain the supplementary crystallographic data for this paper.^40*a*,*b*^ The data supporting this article have been included as part of the supplementary information (SI). Supplementary information: experimental procedures; kinetic data; analyses; X-ray crystallographic data of *endo*-BCN-CH_2_OH 7 and DBCO-acid 10; and computational studies and cartesian coordinates. See DOI: https://doi.org/10.1039/d5sc04275e.
