# Supplementary material for: Temperature–dependent reaction rates of quinone-alkene cycloaddition reveal that only entropy determines the rate of SPOCQ reactions
Source: Chem Sci. 2025 Oct 29;16(48):23357–65. doi: 10.1039/d5sc04275e (PMC12594102; doi:10.1039/d5sc04275e)
Supplement: SC-016-D5SC04275E-s001 [file SC-016-D5SC04275E-s001.pdf]

Supplementary Information (SI) for Chemical Science.
This journal is © The Royal Society of Chemistry 2025

Supporting Information

for

Temperature-Dependent Reaction Rates of Quinone-Alkene Cycloaddition Reveal that Only Entropy Determines the Rate of SPOCQ Reactions

Table of Contents:

1. General information S3
    1.1 Instruments S3
    1.2 General chemicals S3
    1.3 Single crystals for XRD analysis S3
2. Computational details S4
3. References S14
4. Appendices
    Appendix A: Raw data table k_2 – plots and Eyring plots S15
    Appendix B: k_2 - plot experiments S16–S21
    Appendix C: Eyring plot experiments & S22–S28
    Appendix D: Kinetics Studies in MeOH S29–S30
    Appendix E: Fluorescence spectroscopy kinetic studies S31
    Appendix F: XRD of *endo*-BCN-OH S32–S43
    Appendix G: XRD of DBCO-acid S44–S53
    Appendix H: k' data of k_2 plots S54–S57
    Appendix I: k' data of Eyring plots S58–S61
    Appendix J: k' data of SPAAC Fluorescence spectroscopy S62
    Appendix K: Heats of hydrogenation dienophiles S63
    Appendix L: Calculated balance of the entropies
        and enthalpies of activation S64

1. General information

1.1 Instruments

Stopped-flow UV-Vis spectra were recorded on a Cary 60 UV-Vis spectrophotometer (Agilent) equipped with a RX2000 Rapid Kinetics Spectrometer Accessory (Applied Photophysics), which was attached to a Lauda RCS 6-D thermostatic water bath. During UV-Vis experiments, the temperature was measured internally in in RX2000 Accessory, as well as externally in the water bath by the thermostat, along with an external third independent thermometer. Fluorescence measurements were performed on a Edinburgh Instruments FLS900 fluorescence spectrometer equipped with a 450 W xenon lamp and PMT detector. Deionised water was produced with a Milli-Q Integral 3 system (Millipore, Molsheim/France).

1.2 General chemicals

4-*tert*-butyl-*ortho*-quinone **1** was synthesized by the method described by Borrmann.[1] 3-Azido-7-hydroxycoumarin **11** was purchased at TCI. ((1R,8S,9s,E)-bicyclo[6.1.0]non-4-en-9-yl)methanol (*endo*-sTCO-CH_2OH) **2** ; ((1R,8S,9r,E)-bicyclo[6.1.0]non-4-en-9-yl)methanol (*exo*-sTCO-CH_2OH) **3** ; ethyl (1R,8S,9s,E)-bicyclo[6.1.0]non-4-ene-9-carboxylate (*endo*-sTCO-C(O)OEt) **4** and ethyl (1R,8S,9r,E)-bicyclo[6.1.0]non-4-ene-9-carboxylate (*exo*-sTCO-C(O)OEt) **5** were purchased from Synvenio (Nijmegen, The Netherlands). *Trans*-cyclooct-4-enol (TCO-OH) **6** was purchased as a mixture of diastereomers from BroadPharm (San Diego, CA, United States). The potassium salt of DBCO-acid **10** was also purchased from BroadPharm (San Diego, CA, United States). *Exo*-bicyclo[6.1.0]non-4-yn-9-ol (*exo*-BCN-CH_2OH) **8** was purchased from Sirius Fine Chemicals SiChem (Bremen, Germany). *Endo*-bicyclo[6.1.0]non-4-yn-9-ol (*endo*-BCN-CH_2OH) **7** was kindly donated by Synaffix (Oss, The Netherlands). Methanol (CHROMASOLV™) HPLC grade, Riedel-de Haën™ and MilliQ deionised water were specifically used for UV-Vis spectroscopy experiments and for fluorescence spectroscopy experiments.

1.3 Single crystals for XRD analysis

Single crystals of *endo*-BCN-OH (**7**) suitable for X-ray crystallography analysis were grown from a hot Et_2O solution. Single crystals of DBCO-acid (**10**) suitable for X-ray crystallography analysis were grown from a hot THF solution. The structures can be retrieved from the Cambridge Crystallographic Data Centre (CDCC deposition numbers **2378937** and **2378938**). Figures for publication were generated with Olex2.[2]

2. Computational details

All optimizations were performed using Gaussian 16.[3] Geometry optimizations of the minima and transition states were carried out using the M06-2X functional with the 6-311+G(d,p) basis set. Vibrational frequencies were computed at the same level to verify that optimized structures are stationary points, and to evaluate zero-point vibrational energies (ZPVE) and thermal corrections at 298 K. Single-point calculations at the wB97M-V/def2-TZVPP level of theory was performed (using the PSI4 program8), for optimal calculation of the ring strain.

Method: We defined ring strain as the driving force ΔG of the following isodesmic reaction:

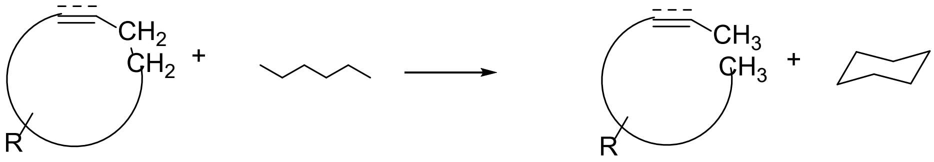

In this way on the left a CH_2-CH_2 bond is hydrogenated, to relieve the ring strain to yield the ring-opened analogue. To makes sure that only ring strain is measured, and not the energy of hydrogenation, a comparison is made with a ring and ring-opened analogue that are both without ring strain, the best case thereof being cyclohexane and n-hexane.

Table S1. Energies of ring-strained compound.

Compound		Free energy (Hartree)	
	M06-2X/6-311+G(d.p)<br>**Electronic energy**<br>**Free energy**<br>**Correction to Gibbs**	Electronic energy wB97M-V/def2-QZVPP //M06-2X/6-311+G(d.p)	wB97M-V/def2-QZVPP //M06-2X/6-311+G(d.p)
Cyclohexane<br>24107	-235.816766601<br>**-235.674265**<br>**0.142501**	-235.8404012	-235.697900
endo-BCN-CH_2OH<br>24101	-464.528927144<br>**-464.343698**<br>**0.185230**	-464.603964	-464.418734
exo-BCN-CH_2OH<br>24110	-464.532369416<br>**-464.347620**<br>**0.184749**	-464.607453	-464.422704
THS<br>24112	-919.197985666<br>**-918.979695**<br>**0.218291**	-919.341489	-919.123198
TCO-OH<br>24111	-388.392505158<br>**-388.215273**<br>**0.177233**	-388.451408	-388.274175
endo-sTCO-CH_2OH<br>24113	-465.761838326<br>**-465.553358**<br>**0.208480**	-465.832663	-465.624183
exo-sTCO-CH_2OH<br>24116	-465.765404132<br>**-465.557129**<br>**0.208275**	-465.836320	-465.628045
endo-sTCO-C(O)OEt<br>24114	-618.415218279<br>**-618.172461**<br>**0.242757**	-618.522454	-618.279697
exo-sTCO-C(O)OEt<br>24115	-618.419186588<br>**-618.177220**<br>**0.241967**	-618.526442	-618.284475
ortho-quinone<br>Hz24129	-538.628742443<br>**-538.467293**<br>**0.161450**	-538.7247756	-538.5631946

Table S2. Energies of ring-opened compounds.

Compound		Free energy (Hartree)	
	M06-2X/6-311+G(d.p)<br>Electronic energy<br>Free energy<br>Correction to Gibbs	Electronic energy wB97M-V/def2-QZVPP //M06-2X/6-311+G(d.p)	wB97M-V/def2-QZVPP //M06-2X/6-311+G(d.p)
n-Hexane<br>24106	-237.010796010<br>**-236.853517**<br>**0.157279**	-237.028944	-236.871665
endo-BCN-CH_2OH<br>24105	-465.744626933<br>**-465.545909**<br>**0.198718**	-465.813327	-465.614609
exo-BCN-CH_2OH<br>24120	-465.748115938<br>**-465.550520**<br>**0.197596**	-465.817223	-465.619627
TCO-OH<br>24121	-389.614093279<br>**-389.424108**<br>**0.189986**	-389.667257	-389.477271
THS<br>24122	-920.417006548<br>**-920.184746**<br>**0.232261**	-920.554707	-920.322446
endo-sTCO-CH_2OH<br>24123	-466.982997381<br>**-466.760953**<br>**0.222045**	-467.048226	-466.826181
exo-sTCO-CH_2OH<br>24126	-466.985876545<br>**-466.763834**<br>**0.222042**	-467.051354	-466.829312
endo-sTCO-C(O)OEt<br>24124	-619.636649171<br>**-619.380659**<br>**0.255990**	-619.738555	-619.482565
exo-sTCO-C(O)OEt<br>24125	-619.640109464<br>**-619.384466**<br>**0.255644**	-619.742035	-619.486391

Table S3. Comparison of ring strain energies at different computational levels.

Compound	Ring strain (kcal/mol)	
	**M06-2X/6-311+G(d,p)**	**wB97M-V/def2-QZVPP //M06-2X/6-311+G(d,p)**
endo-BCN-CH_2OH	**14.41**	**13.87**
exo-BCN-CH_2OH	**14.84**	**14.53**
THS	**16.19**	**15.94**
TCO-OH	**18.56**	**18.41**
endo-sTCO-CH_2OH	**17.78**	**17.72**
exo-sTCO-CH_2OH	**17.23**	**17.25**
endo-sTCO-C(O)OEt	**18.16**	**18.26**
exo-sTCO-C(O)OEt	**17.57**	**17.60**

Cartesian coordinates of optimized compounds at M06-2X/6-311+G(d,p) level of theory.

Cyclic structures

***ortho*-quinone**

6	0.300432000	1.059873000	-0.000012000
6	1.402465000	-1.628807000	-0.000006000
6	1.751225000	0.919366000	-0.000033000
6	2.347217000	-0.513305000	-0.000029000
6	-0.503040000	-0.022771000	0.000001000
6	0.085427000	-1.384054000	-0.000010000
1	-0.079701000	2.073605000	-0.000003000
8	2.519838000	1.859975000	0.000013000
8	3.552086000	-0.645059000	0.000064000
1	1.807938000	-2.633547000	0.000003000
1	-0.599541000	-2.223805000	-0.000027000
6	-2.018843000	0.063648000	-0.000002000
6	-2.512579000	1.510184000	-0.000244000
1	-2.173502000	2.049807000	-0.888164000
1	-3.604773000	1.511778000	-0.000189000
1	-2.173408000	2.050132000	0.887444000
6	-2.554733000	-0.641714000	1.258866000
1	-2.177162000	-0.157553000	2.163128000
1	-3.645824000	-0.577634000	1.263704000
1	-2.281751000	-1.698479000	1.290819000
6	-2.554730000	-0.642143000	-1.258628000
1	-3.645827000	-0.578164000	-1.263436000
1	-2.177241000	-0.158230000	-2.163056000
1	-2.281645000	-1.698894000	-1.290260000

***endo*-sTCO-CH_2OH**

6	0.104795000	-0.927318000	0.884790000
6	0.405721000	0.564772000	0.900430000
6	0.056066000	1.546099000	-0.209042000
6	-1.406502000	2.064515000	-0.089561000
6	-2.214571000	0.831214000	0.204223000
6	-2.307423000	-0.151973000	-0.689229000
6	-2.110994000	-1.575667000	-0.268206000
6	-0.563884000	-1.733333000	-0.222658000
1	-1.697035000	2.543421000	-1.028274000
1	-1.488764000	2.803437000	0.710488000
1	0.150334000	1.057096000	-1.181932000
1	0.762222000	2.383097000	-0.193333000
1	-0.161348000	-1.305053000	1.868374000
1	-0.170682000	-1.456630000	-1.204337000
1	-0.312001000	-2.788253000	-0.070738000
1	-2.530327000	-1.758971000	0.725617000
1	-2.531059000	-2.303344000	-0.965823000
1	-2.085909000	0.068276000	-1.734788000
1	-2.361599000	0.584856000	1.254580000
1	0.292727000	1.001869000	1.889286000
6	1.534297000	-0.432798000	0.801017000
1	2.138108000	-0.566727000	1.693125000
6	2.330774000	-0.567557000	-0.464681000
1	2.816991000	-1.548429000	-0.499235000
1	1.704376000	-0.468587000	-1.353323000
8	3.328806000	0.462386000	-0.467365000
1	3.873855000	0.347126000	-1.253272000

***exo*-sTCO-CH_2OH**

6	0.248805000	-0.916693000	0.335646000
6	0.502058000	0.582660000	0.253745000
6	-0.285030000	1.564993000	-0.598491000
6	-1.596449000	2.040284000	0.089671000
6	-2.200075000	0.780490000	0.645616000
6	-2.609019000	-0.192315000	-0.166746000
6	-2.231667000	-1.615226000	0.107115000
6	-0.781513000	-1.720448000	-0.444345000
1	-2.239036000	2.521573000	-0.652230000
1	-1.378035000	2.767329000	0.874844000
1	-0.558542000	1.078963000	-1.539826000
1	0.351898000	2.420802000	-0.846450000
1	0.424989000	-1.313776000	1.332872000
1	-0.786407000	-1.399913000	-1.491378000
1	-0.465906000	-2.769102000	-0.439783000
1	-2.234556000	-1.829973000	1.179954000
1	-2.869087000	-2.344282000	-0.397578000
1	-2.814214000	0.050480000	-1.210603000
1	-1.921977000	0.515499000	1.664452000
1	0.822325000	1.000786000	1.206347000
6	1.486264000	-0.384298000	-0.344127000
1	1.474653000	-0.449333000	-1.429668000
6	2.830813000	-0.571083000	0.289993000
1	2.723996000	-0.580074000	1.380235000
1	3.277789000	-1.521126000	-0.021086000
8	3.680776000	0.511514000	-0.108353000
1	4.560786000	0.349846000	0.248254000

***endo*-sTCO-C(O)OEt**

6	0.807752000	0.673531000	1.061655000
6	0.833250000	-0.824303000	0.946854000
6	1.268799000	-1.620634000	-0.273756000
6	2.812498000	-1.817664000	-0.290667000
6	3.362865000	-0.459160000	0.050292000
6	3.147013000	0.581863000	-0.751903000
6	2.689615000	1.891764000	-0.187354000
6	1.156111000	1.697129000	-0.009132000

1	3.116502000	-2.158526000	-1.283526000
1	3.116128000	-2.571896000	0.438198000
1	0.994308000	-1.089005000	-1.184883000
1	0.751745000	-2.585440000	-0.282358000
1	1.063005000	1.024784000	2.057273000
1	0.732502000	1.412408000	-0.973865000
1	0.696464000	2.649214000	0.274082000
1	3.154425000	2.092306000	0.782413000
1	2.870520000	2.743157000	-0.846471000
1	2.873974000	0.393644000	-1.791368000
1	3.558911000	-0.263739000	1.103496000
1	1.107470000	-1.314086000	1.877249000
6	-0.506588000	-0.111894000	1.079185000
1	-1.003991000	-0.191533000	2.035760000
6	-1.451496000	-0.042469000	-0.051855000
8	-1.179929000	0.008266000	-1.235003000
8	-2.721516000	-0.042947000	0.385601000
6	-3.754446000	0.022546000	-0.621945000
1	-3.614270000	0.936137000	-1.201995000
1	-3.644520000	-0.835622000	-1.286923000
6	-5.082413000	0.010950000	0.094890000
1	-5.169703000	0.869582000	0.762998000
1	-5.888622000	0.060979000	-0.639056000
1	-5.201035000	-0.904870000	0.676603000

***exo*-sTCO-C(O)OEt**

6	0.704164000	0.721825000	0.370204000
6	0.704246000	-0.775128000	0.233619000
6	1.575978000	-1.586077000	-0.711194000
6	2.986068000	-1.860419000	-0.114984000
6	3.407191000	-0.538327000	0.465583000
6	3.591121000	0.519477000	-0.322068000
6	3.000038000	1.846539000	0.041737000
6	1.522511000	1.726482000	-0.426778000
1	3.650609000	-2.200378000	-0.913218000
1	2.937409000	-2.641890000	0.645955000
1	1.707274000	-1.028795000	-1.642879000
1	1.067611000	-2.523727000	-0.955904000
1	0.484343000	1.047764000	1.383236000
1	1.515461000	1.453928000	-1.486794000
1	1.030061000	2.700416000	-0.346564000
1	3.032618000	2.016457000	1.121860000
1	3.472292000	2.692724000	-0.461149000
1	3.765203000	0.354905000	-1.386542000
1	3.157103000	-0.363914000	1.510976000
1	0.486127000	-1.278460000	1.172016000
6	-0.471651000	0.021543000	-0.297334000
1	-0.549427000	0.125558000	-1.372484000
6	-1.748462000	-0.047432000	0.432974000
8	-1.856544000	-0.164661000	1.639404000
8	-2.806494000	0.034076000	-0.380569000
6	-4.110988000	-0.022630000	0.240633000
1	-4.192791000	-0.964834000	0.784686000
1	-4.190032000	0.801868000	0.950904000
6	-5.137551000	0.082289000	-0.859951000
1	-5.035280000	-0.743821000	-1.565760000
1	-6.137418000	0.042735000	-0.424390000
1	-5.032849000	1.025297000	-1.399285000

***endo*-BCN-CH_2OH**

6	-2.514319000	-0.144423000	-0.259067000
6	-2.233393000	1.026703000	-0.198636000
6	-2.212460000	-1.579237000	-0.271143000
6	-0.666942000	-1.658648000	-0.354588000
1	-0.360470000	-1.221210000	-1.306760000
1	-0.367920000	-2.711726000	-0.366027000
6	0.010777000	-0.963812000	0.814979000
6	0.355336000	0.511748000	0.910678000
6	0.100781000	1.561570000	-0.156755000
1	0.228923000	1.142374000	-1.157412000
1	0.832364000	2.368024000	-0.044706000
6	-1.323033000	2.168219000	-0.066960000
1	-1.471433000	2.910177000	-0.853648000
1	-1.466277000	2.671289000	0.893064000
1	-2.575299000	-2.062291000	0.640016000
1	-2.667226000	-2.091741000	-1.120763000
6	1.455061000	-0.519427000	0.789600000
1	-0.294485000	-1.388967000	1.767309000
1	0.236435000	0.911205000	1.914282000
1	2.030423000	-0.714144000	1.688940000
6	2.274271000	-0.621250000	-0.464552000
1	1.661159000	-0.508930000	-1.361001000
1	2.764899000	-1.599187000	-0.510253000
8	3.267767000	0.411959000	-0.429880000
1	3.830294000	0.310790000	-1.205345000

***exo*-BCN-CH_2OH**

6	2.623480000	-0.279946000	-0.290471000
6	2.417904000	0.908246000	-0.289995000
6	2.260517000	-1.687863000	-0.103373000
6	0.859731000	-1.654732000	0.557418000
1	0.950853000	-1.182344000	1.539217000
1	0.522462000	-2.683307000	0.720476000
6	-0.176183000	-0.924874000	-0.277368000
6	-0.438158000	0.572048000	-0.276409000
6	0.292530000	1.608904000	0.555723000
1	0.536128000	1.196831000	1.538885000
1	-0.371626000	2.464134000	0.716773000
6	1.601367000	2.111409000	-0.104022000
1	2.090765000	2.850393000	0.533062000

1	1.388941000	2.591145000	-1.063165000
1	2.221256000	-2.212005000	-1.061982000
1	2.970908000	-2.218053000	0.533592000
6	-1.427291000	-0.373794000	0.352837000
1	-0.330732000	-1.379986000	-1.253180000
1	-0.745534000	0.946994000	-1.250547000
1	-1.434379000	-0.378364000	1.440293000
6	-2.757998000	-0.605978000	-0.294779000
1	-2.631483000	-0.666398000	-1.381219000
1	-3.199775000	-1.545155000	0.054423000
8	-3.625912000	0.484546000	0.036190000
1	-4.495871000	0.299234000	-0.333513000

THS

6	-1.895984000	-0.812634000	0.002420000
6	1.905937000	-0.794063000	0.004694000
6	-1.505806000	0.524297000	-0.679735000
1	-2.287453000	1.280175000	-0.552711000
1	-1.323000000	0.378788000	-1.746889000
6	1.502516000	0.555030000	-0.649115000
1	2.272404000	1.318609000	-0.502805000
1	1.330141000	0.427940000	-1.720495000
6	-0.594572000	-1.491759000	0.143232000
6	0.610713000	-1.483894000	0.147279000
6	-2.559970000	-0.586078000	1.364920000
1	-2.833520000	-1.547452000	1.803955000
1	-1.895158000	-0.066239000	2.055194000
1	-3.468149000	0.006536000	1.230249000
6	-2.859178000	-1.552967000	-0.931303000
1	-3.752629000	-0.945467000	-1.094386000
1	-2.390359000	-1.757438000	-1.895528000
1	-3.159510000	-2.498805000	-0.476159000
6	2.592394000	-0.593152000	1.359228000
1	2.860225000	-1.563982000	1.780798000
1	3.506662000	-0.011582000	1.216273000
1	1.942350000	-0.066502000	2.056338000
6	2.856193000	-1.511054000	-0.960879000
1	3.741751000	-0.893864000	-1.130658000
1	3.172473000	-2.461452000	-0.526810000
1	2.370667000	-1.703762000	-1.919262000
16	-0.004348000	1.414646000	-0.100779000
8	-0.042370000	2.644336000	-0.949809000
7	0.110188000	1.552156000	1.431817000
1	-0.763139000	1.928022000	1.806669000

TCO-OH

6	1.127361000	-0.436544000	-0.233051000
6	0.089173000	-1.242876000	0.604936000
6	-1.263248000	-1.567804000	-0.063366000
6	-2.306936000	-0.420488000	-0.009885000
6	-1.581420000	0.783487000	-0.515727000
6	-0.851387000	1.537609000	0.304939000
6	0.483399000	2.071811000	-0.104511000
6	1.497042000	0.966589000	0.279275000
1	-2.620797000	-0.269186000	1.027221000
1	-3.183220000	-0.692689000	-0.602550000
1	-1.691450000	-2.451576000	0.418245000
1	-1.085005000	-1.836500000	-1.110489000
1	-0.083830000	-0.746664000	1.564915000
1	0.566128000	-2.198503000	0.842346000
1	0.762341000	-0.350208000	-1.263953000
1	1.601155000	0.927054000	1.368897000
1	2.477117000	1.223729000	-0.131834000
1	0.514721000	2.231638000	-1.186122000
1	0.759084000	3.004471000	0.392489000
1	-1.035247000	1.470133000	1.377361000
1	-1.360441000	0.804980000	-1.583177000
8	2.377249000	-1.151966000	-0.265952000
1	2.197550000	-2.051659000	-0.561401000

Open structures

Open structure for *endo*-sTCO-CH_2OH

6	-1.308784000	-0.520404000	-0.895128000
6	-0.237821000	-1.121216000	0.025956000
6	1.116485000	-1.194287000	-0.644720000
6	2.410605000	-1.043842000	0.112537000
6	2.460229000	-0.787028000	1.601954000
6	-4.715845000	0.594033000	0.682057000
6	-3.367509000	0.583496000	0.026042000
6	-2.667152000	-0.510611000	-0.258809000
6	1.985398000	0.026967000	-0.861431000
6	1.579409000	1.387424000	-0.370690000
8	2.747366000	2.046556000	0.106113000
1	-1.022107000	0.499565000	-1.171582000
1	-1.337395000	-1.102181000	-1.824193000
1	-0.539983000	-2.132566000	0.320576000
1	-0.199131000	-0.540753000	0.950429000
1	1.143492000	-1.905112000	-1.465332000
1	3.218848000	-1.673801000	-0.242878000
1	2.290511000	-1.710828000	2.161314000
1	3.438288000	-0.389247000	1.878156000
1	1.717543000	-0.058233000	1.926453000
1	-5.461560000	1.068589000	0.038235000
1	-5.051873000	-0.420055000	0.904878000
1	-4.693264000	1.161157000	1.616727000
1	2.538659000	0.058525000	-1.793461000
1	0.829738000	1.315557000	0.424918000
1	1.132067000	1.953934000	-1.196965000
1	2.517351000	2.945936000	0.350046000
1	-3.085056000	-1.483491000	0.001871000
1	-2.945137000	1.553359000	-0.234717000

Open structure for *exo*-sTCO-CH_2OH

6	-1.116762000	-0.911138000	0.401403000
6	-0.456338000	0.464094000	0.464749000
6	1.024608000	0.333806000	0.734034000
6	2.036402000	1.368886000	0.314383000
6	1.643312000	2.645750000	-0.391802000
6	-4.829169000	0.100424000	-0.388068000
6	-3.358910000	0.157583000	-0.093029000
6	-2.590296000	-0.910012000	0.105624000
6	1.972351000	0.042548000	-0.396775000
6	3.049336000	-0.981356000	-0.208844000
8	2.459982000	-2.269371000	-0.338629000
1	-0.596462000	-1.517190000	-0.350169000
1	-0.949282000	-1.434392000	1.351147000
1	-0.927706000	1.070843000	1.246033000
1	-0.621436000	0.986040000	-0.482456000
1	1.259498000	-0.185629000	1.659953000
1	2.871978000	1.482587000	0.999075000
1	2.504307000	3.082134000	-0.903579000
1	0.871416000	2.469096000	-1.142035000
1	1.260906000	3.385823000	0.315880000
1	-5.055433000	0.567425000	-1.350752000
1	-5.184284000	-0.931248000	-0.417306000
1	-5.403705000	0.638833000	0.370917000
1	1.531846000	0.058793000	-1.390646000
1	3.841167000	-0.845907000	-0.956026000
1	3.494693000	-0.858758000	0.787392000
1	3.149992000	-2.934312000	-0.286441000
1	-2.918515000	1.151053000	-0.044313000
1	-3.056044000	-1.893734000	0.052311000

Open structure for *exo*-sTCO-C(O)OEt

6	-0.229606000	1.539420000	-0.040396000
6	0.167465000	0.265855000	0.630435000
6	1.401181000	-0.509321000	0.231694000
6	2.675638000	0.098019000	0.833583000
6	3.898765000	-0.701980000	0.495154000
6	4.920707000	-0.258633000	-0.231167000
6	6.134681000	-1.063525000	-0.586761000
6	0.557155000	2.150260000	-1.175280000
1	2.556339000	0.152739000	1.922099000
1	2.793017000	1.125314000	0.475288000
1	1.501475000	-0.546477000	-0.856571000
1	1.296252000	-1.547450000	0.564443000
1	-0.732330000	2.246701000	0.610680000
1	1.044423000	1.390051000	-1.787948000
1	-0.098024000	2.733364000	-1.825971000
1	6.070954000	-2.072731000	-0.176464000
1	6.250419000	-1.140397000	-1.671370000
1	4.890514000	0.766171000	-0.599514000
1	3.922754000	-1.727421000	0.865075000
1	-0.089474000	0.217309000	1.684418000
6	-1.041948000	0.280715000	-0.283690000
1	-0.946652000	-0.200137000	-1.249227000
6	-2.375048000	0.201263000	0.351359000
8	-2.658122000	0.675500000	1.423159000
8	-3.254095000	-0.470207000	-0.410414000
6	-4.578036000	-0.598415000	0.130248000
1	-4.515324000	-1.107132000	1.094479000
1	-4.983106000	0.400152000	0.307005000
6	-5.399516000	-1.377136000	-0.871971000
1	-4.968786000	-2.365713000	-1.036875000
1	-6.418499000	-1.500010000	-0.500767000
1	-5.439278000	-0.852070000	-1.827339000
1	7.043437000	-0.593274000	-0.201064000
1	1.330991000	2.819540000	-0.791590000

Open structure for *endo*-sTCO-C(O)OEt

6	1.782685000	0.236719000	-1.053180000
6	1.243807000	1.458229000	-0.296355000
6	-0.054188000	1.957709000	-0.889266000
6	-1.138002000	2.589643000	-0.082293000
6	-1.034027000	2.812044000	1.408609000
6	4.670483000	-1.768705000	0.665972000
6	3.342785000	-1.338443000	0.118312000
6	3.120723000	-0.202125000	-0.535383000
6	-1.372026000	1.212716000	-0.693806000
1	1.066341000	-0.585525000	-0.960920000
1	1.857848000	0.480654000	-2.119789000
1	1.976730000	2.271485000	-0.342697000
1	1.123436000	1.190902000	0.753058000
1	0.052128000	2.337502000	-1.901296000
1	-1.697402000	3.362467000	-0.599064000
1	-0.474961000	3.729782000	1.606667000
1	-2.029909000	2.923844000	1.843148000
1	-0.540730000	1.986068000	1.916071000
1	4.985701000	-2.720548000	0.229411000
1	5.441285000	-1.024603000	0.457729000
1	4.617930000	-1.915172000	1.748335000
1	-2.034750000	1.166987000	-1.546774000
1	3.951508000	0.486796000	-0.690804000
1	2.504604000	-2.014986000	0.280417000
6	-1.428724000	-0.002447000	0.149443000
8	-0.792546000	-0.224109000	1.150682000
8	-2.307767000	-0.887978000	-0.356637000
6	-2.440073000	-2.119643000	0.367290000
1	-1.463910000	-2.607913000	0.411113000
1	-2.745026000	-1.895221000	1.391568000
6	-3.464244000	-2.960933000	-0.360382000
1	-4.426358000	-2.448147000	-0.397376000
1	-3.139461000	-3.163596000	-1.381862000
1	-3.597685000	-3.912652000	0.156950000

Open structure for *endo*-BCN-CH_2OH

8	-3.334933000	0.468407000	-0.313397000
6	-2.377134000	-0.576516000	-0.402283000
6	-1.480977000	-0.494951000	0.788333000
6	-0.269564000	0.420635000	0.851587000
6	0.004159000	1.379367000	-0.276749000
6	1.181547000	2.318226000	-0.000402000
6	2.487675000	1.674100000	-0.205028000
6	3.569289000	1.184511000	-0.385172000
6	1.878318000	-2.301525000	-0.153611000
6	0.443737000	-1.849372000	-0.378317000
6	-0.087813000	-1.090863000	0.800928000
1	-3.908185000	0.388855000	-1.093784000
1	-1.842789000	-0.473825000	-1.350542000
1	-2.908465000	-1.533337000	-0.405201000
1	-2.063240000	-0.591534000	1.700707000
1	-0.112666000	0.900666000	1.814744000
1	-0.889343000	2.006618000	-0.391228000
1	0.146548000	0.866560000	-1.232529000
1	1.111149000	3.172467000	-0.684801000
1	1.122340000	2.723193000	1.016411000
1	4.528383000	0.749578000	-0.548411000
1	1.946226000	-2.975757000	0.706655000
1	2.253014000	-2.834607000	-1.032647000
1	2.534990000	-1.450157000	0.040187000
1	0.394262000	-1.267684000	-1.302621000
1	-0.178524000	-2.739412000	-0.531467000
1	0.194191000	-1.580945000	1.730610000

Open structure for *exo*-BCN-CH_2OH

6	-1.197297000	-0.644707000	0.415033000
6	-0.389837000	0.661700000	0.471251000
6	1.068285000	0.369860000	0.729709000
6	2.187419000	1.268494000	0.270166000
6	1.936199000	2.563919000	-0.466146000
6	-5.187018000	0.060011000	-0.373165000
6	-3.775452000	-0.194800000	-0.093067000
6	-2.615526000	-0.414976000	0.136523000
6	1.959248000	-0.058258000	-0.404286000
6	2.905589000	-1.201166000	-0.200107000
8	2.155914000	-2.407538000	-0.274545000
1	-0.766106000	-1.298516000	-0.349428000
1	-1.094922000	-1.177329000	1.365739000
1	-0.799092000	1.308224000	1.253356000
1	-0.516618000	1.191536000	-0.475716000
1	1.254555000	-0.149887000	1.666499000
1	3.039069000	1.300969000	0.943484000
1	2.835650000	2.882674000	-0.998041000
1	1.139210000	2.459686000	-1.203555000
1	1.652101000	3.361376000	0.225087000
1	-5.371436000	1.129242000	-0.492428000
1	-5.496550000	-0.442399000	-1.291439000
1	-5.815517000	-0.303819000	0.441757000
1	1.508686000	-0.014974000	-1.392873000
1	3.691060000	-1.189852000	-0.965715000
1	3.384738000	-1.103762000	0.783135000
1	2.758210000	-3.153333000	-0.228964000

Open structure for THS

6	-0.004043000	-0.670023000	0.181965000
6	3.879056000	0.736616000	-0.163359000
6	-0.918332000	0.575870000	0.146060000
1	-0.632447000	1.235552000	-0.675234000
1	-0.851351000	1.115142000	1.093896000

6	-3.387566000	1.831367000	0.330058000
1	-4.442095000	1.771838000	0.065333000
1	-2.886910000	2.608201000	-0.245522000
6	1.371954000	-0.159843000	0.057327000
6	2.495453000	0.261185000	-0.044461000
6	-0.129456000	-1.409197000	1.526632000
1	0.545563000	-2.266712000	1.534500000
1	0.147907000	-0.748831000	2.350705000
1	-1.149991000	-1.753705000	1.692301000
6	-0.290229000	-1.625714000	-0.990176000
1	-1.269482000	-2.097748000	-0.882848000
1	-0.279052000	-1.093737000	-1.941965000
1	0.470059000	-2.407840000	-1.008835000
6	4.614460000	0.602475000	1.176368000
1	4.666624000	-0.447792000	1.471999000
1	5.632665000	0.987479000	1.086265000
1	4.100538000	1.154478000	1.964333000
6	4.615344000	-0.020418000	-1.276408000
1	5.633171000	0.361948000	-1.380859000
1	4.668492000	-1.084276000	-1.034399000
1	4.101080000	0.087655000	-2.232096000
16	-2.696674000	0.255211000	-0.142790000
8	-2.832396000	0.081089000	-1.597243000
7	-3.360390000	-0.710722000	0.854797000
1	-3.180320000	-1.676414000	0.588289000
1	3.841543000	1.797656000	-0.433206000
1	-3.267159000	1.966180000	1.402318000

Open structure for TCO

6	1.065818000	-0.705869000	0.549764000
6	-0.127196000	0.119261000	0.053908000
6	-1.448559000	-0.629515000	0.207417000
6	-2.636031000	0.072235000	-0.435192000
6	-2.921126000	1.440294000	0.170063000
6	4.684738000	0.467602000	-0.381107000
6	3.399628000	-0.298967000	-0.284716000
6	2.358687000	0.050772000	0.465189000
8	-3.746697000	-0.801060000	-0.256072000
1	0.878714000	-1.000319000	1.589587000
1	1.141281000	-1.629725000	-0.032311000
1	0.036600000	0.382156000	-0.997713000
1	-0.160429000	1.062216000	0.610081000
1	-1.676595000	-0.783556000	1.268285000
1	-1.366724000	-1.622882000	-0.245075000
1	-2.433954000	0.188815000	-1.509935000
1	-3.827019000	1.873813000	-0.263241000
1	-3.066462000	1.347878000	1.249559000
1	-2.101339000	2.136134000	-0.018744000
1	5.532058000	-0.146079000	-0.062909000
1	4.882055000	0.774746000	-1.411858000
1	4.656441000	1.362019000	0.243580000
1	3.330648000	-1.210483000	-0.877174000
1	2.422537000	0.963560000	1.058452000
1	-4.529983000	-0.384699000	-0.623959000

3. References

(1) Borrmann, A.; Fatunsin, O.; Dommerholt, J.; Jonker, A. M.; Löwik, D. W. P. M.; Van Hest, J. C. M.; Van Delft, F. L. Strain-promoted oxidation-controlled cyclooctyne-1,2-quinone cycloaddition (SPOCQ) for fast and activatable protein conjugation. *Bioconjugate Chem*. **2015**, *26*, 257–261.

(2) Dolomanov, O.V.; Bourhis, L.J.; Gildea, R.J.; Howard, J.A.K.; Puschmann, H. OLEX2: A complete structure solution, refinement and analysis program. *J. Appl. Cryst.* **2009**, *42*, 339–341.

(3) Gaussian 16, Revision B.01, M. J. Frisch, G. W. Trucks, H. B. Schlegel, G. E. Scuseria, M. A. Robb, J. R. Cheeseman, G. Scalmani, V. Barone, G. A. Petersson, H. Nakatsuji, X. Li, M. Caricato, A. V. Marenich, J. Bloino, B. G. Janesko, R. Gomperts, B. Mennucci, H. P. Hratchian, J. V. Ortiz, A. F. Izmaylov, J. L. Sonnenberg, D. Williams-Young, F. Ding, F. Lipparini, F. Egidi, J. Goings, B. Peng, A. Petrone, T. Henderson, D. Ranasinghe, V. G. Zakrzewski, J. Gao, N. Rega, G. Zheng, W. Liang, M. Hada, M. Ehara, K. Toyota, R. Fukuda, J. Hasegawa, M. Ishida, T. Nakajima, Y. Honda, O. Kitao, H. Nakai, T. Vreven, K. Throssell, J. A. Montgomery, Jr., J. E. Peralta, F. Ogliaro, M. J. Bearpark, J. J. Heyd, E. N. Brothers, K. N. Kudin, V. N. Staroverov, T. A. Keith, R. Kobayashi, J. Normand, K. Raghavachari, A. P. Rendell, J. C. Burant, S. S. Iyengar, J. Tomasi, M. Cossi, J. M. Millam, M. Klene, C. Adamo, R. Cammi, J. W. Ochterski, R. L. Martin, K. Morokuma, O. Farkas, J. B. Foresman, and D. J. Fox, Gaussian, Inc., Wallingford CT, **2016**.

(4) Psi4 1.4: Open-Source Software for High-Throughput Quantum Chemistry", D. G. A. Smith, L. A. Burns, A. C. Simmonett, R. M. Parrish, M. C. Schieber, R. Galvelis, P. Kraus, H.

Kruse, R. Di Remigio, A. Alenaizan, A. M. James, S. Lehtola, J. P. Misiewicz, M. Scheurer, R. A. Shaw, J. B. Schriber, Y. Xie, Z. L. Glick, D. A. Sirianni, J. S. O'Brien, J. M. Waldrop, A. Kumar, E. G. Hohenstein, B. P. Pritchard, B. R. Brooks, H. F. Schaefer III, A. Yu. Sokolov, K. Patkowski, A. E. DePrince III, U. Bozkaya, R. A. King, F. A. Evangelista, J. M. Turney, T. D. Crawford, C. D. Sherrill, J. Chem. Phys. (2020). (doi: 10.1063/5.0006002).

4. Appendices

Appendix A: Raw data table k_2 – plots and Eyring plots

Table A1: Unrounded numerical values of the thermodynamic activation parameters and second order rate constants for the inverse electron-demand Diels-Alder SPOCQ cycloadditions between 4-*tert*-butyl-1,2-*ortho*-quinone **1** and different strained dienophiles **2–9**. Standard deviations are given between the brackets.

	Eyring plot					k_2 plot[b]	
dienophile	R^2	$\Delta H^‡$ (kcal/mol)	$\Delta S^‡$ (cal/K·mol)	$T\Delta S^{‡,b}$ (kcal/mol)	$\Delta G^{‡,b}$ (kcal/mol)	R^2	k_2 ($M^{-1}s^{-1}$)
cycloalkenes							
endo-sTCO-CH_2OH (**2**)	0.9610	0.75 (±0.20)	–39.98 (±0.67)	–11.92	12.67	0.9995	3354 (±52)
exo-sTCO-CH_2OH (**3**)	0.9924	0.68 (±0.10)	–39.80 (±0.36)	–11.87	12.54	0.9996	3525 (±52)
endo-sTCO-C(O)OEt (**4**)	0.9844	1.05 (±0.18)	–39.90 (±0.64)	–11.90	12.95	0.9945	1868 (±78)
exo-sTCO-C(O)OEt (**5**)	0.9976	1.12 (±0.07)	–41.67 (±0.24)	–12.42	13.55	0.9979	818 (±22)
TCO-OH (**6**)[a]	0.9774	0.54 (±0.12)	–51.93 (±0.40)	–15.48	16.02	0.9997	11.56 (±0.11)
TCO-OH axial (**6-ax**)	0.9725	2.07 (±0.23)	–44.40 (±0.28)	–13.24	15.30	n.d.	34.85
cycloalkynes							
endo-BCN-CH_2OH (**7**)[a]	0.9908	2.25 (±0.27)	–36.30 (±0.94)	–10.82	13.07	0.9998	1824 (±16)
exo-BCN-CH_2OH (**8**)	0.9603	1.73 (±0.45)	–38.32 (±1.54)	–11.43	13.15	0.9999	1684 (±11)
THS (**9**)[a]	0.9747	0.80 (±0.16)	–46.85 (±0.56)	–13.97	14.77	0.9987	110.6 (±2.3)

Notes: [a] these values were reported previously [Damen, *Chem. Eur. J.*, **2023**, e202300231; ref 17 in the manuscript]. [b] determined at 25 °C.

***Appendix B*: k_2 - plot experiments**

B.1. k_2 plot *endo*-sTCO-CH_2OH **2**

Stoichiometry	Initial concentration *o*-quinone [mM]	Final concentration *o*-quinone [mM]	Initial concentration probe [mM]	Final concentration probe [mM]	*k'* (s^{-1})
1 vs 10	0.0968	0.0484	0.966	0.483	1.551
1 vs 25	0.0968	0.0484	2.42	1.21	4.143
1 vs 50	0.0968	0.0484	4.84	2.42	7.973
1 vs 75	0.0968	0.0484	7.29	3.65	12.25
1 vs 100	-	-	-	-	-

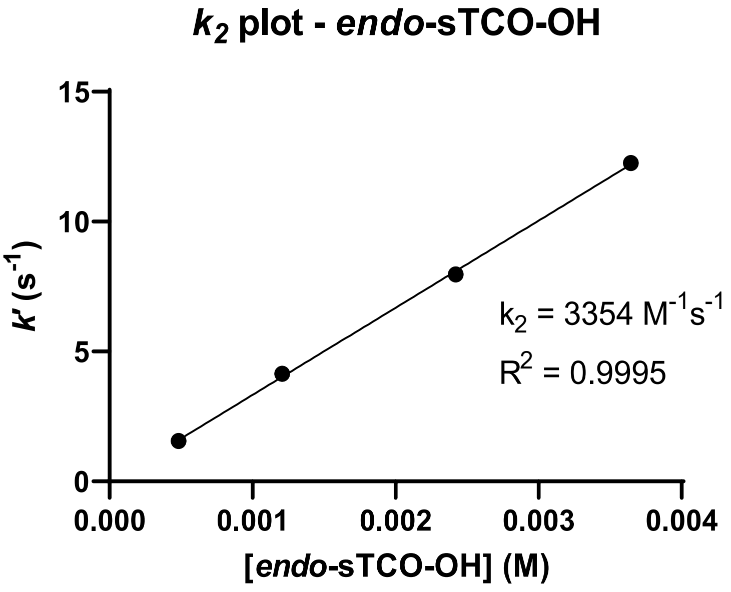

Best-fit values
  Slope 3354
  Y-intercept -0.02642
  X-intercept 7.875e-006
  1/slope 0.0002981

Std. Error
  Slope 52.36
  Y-intercept 0.1195

Goodness of Fit
  R squared 0.9995
  Sy.x 0.1261

Equation Y = 3354*X - 0.02642

B.2. k_2 plot *exo*-sTCO-CH_2OH **3**

Stoichiometry	Initial concentration *o*-quinone [mM]	Final concentration *o*-quinone [mM]	Initial concentration probe [mM]	Final concentration probe [mM]	*k'* (s^{-1})
1 vs 10	0.0980	0.0490	0.979	0.489	1.687
1 vs 25	0.0980	0.0490	2.45	1.22	4.436
1 vs 50	0.0980	0.0490	4.89	2.45	8.514
1 vs 75	0.0980	0.0490	7.34	3.67	12.99
1 vs 100	-	-	-	-	-

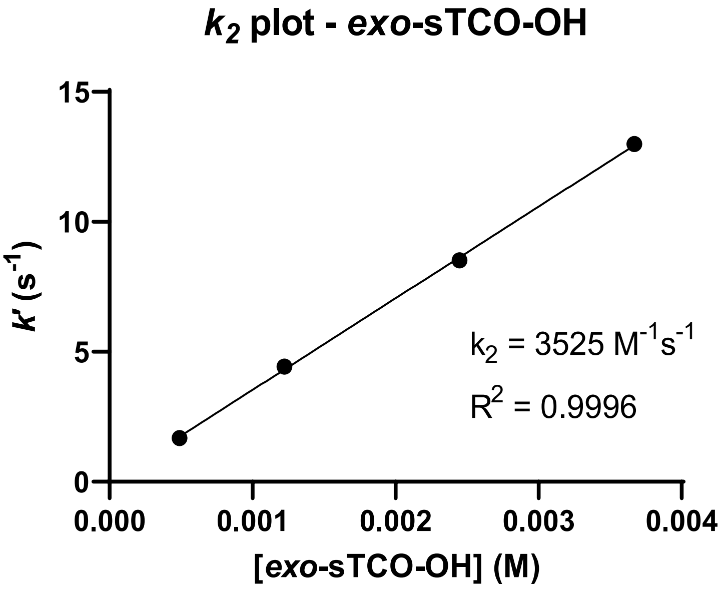

Best-fit values

Slope	3525
Y-intercept	0.006513
X-intercept	-1.848e-006
1/slope	0.0002837

Std. Error

Slope	51.87
Y-intercept	0.1194

Goodness of Fit

R squared	0.9996
Sy.x	0.1256

Equation Y = 3525*X + 0.006513

B.3. k_2 plot *endo*-sTCO-C(O)OEt **4**

Stoichiometry	Initial concentration *o*-quinone [mM]	Final concentration *o*-quinone [mM]	Initial concentration probe [mM]	Final concentration probe [mM]	*k'* (s^{-1})
1 vs 10	0.0743	0.0371	0.741	0.371	0.7464
1 vs 25	0.0743	0.0371	1.86	0.93	1.922
1 vs 50	0.0743	0.0371	3.72	1.86	3.855
1 vs 75	0.0743	0.0371	5.59	2.79	5.610
1 vs 100	0.0743	0.0371	7.41	3.71	6.900

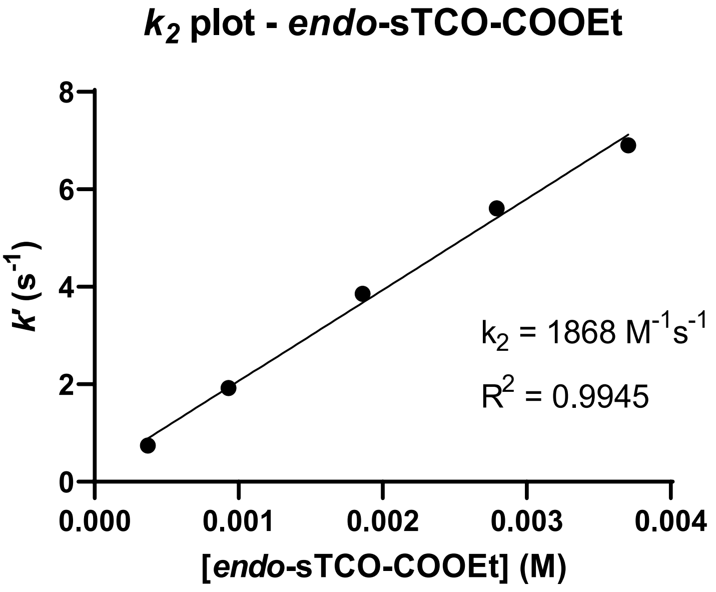

Best-fit values
Slope 1868
Y-intercept 0.1982
X-intercept -0.0001061
1/slope 0.0005354

Std. Error
Slope 79.98
Y-intercept 0.1824

Goodness of Fit
R squared 0.9945
Sy.x 0.2166

Equation Y = 1868*X + 0.1982

B.4. k_2 plot *exo*-sTCO-C(O)OEt **5**

Stoichiometry	Initial concentration *o*-quinone [mM]	Final concentration *o*-quinone [mM]	Initial concentration probe [mM]	Final concentration probe [mM]	*k'* (s^{-1})
1 vs 10	0.0786	0.0393	0.788	0.394	0.2831
1 vs 25	0.0786	0.0393	1.97	0.98	0.7609
1 vs 50	0.0786	0.0393	3.93	1.96	1.445
1 vs 75	0.0786	0.0393	5.89	2.95	2.339
1 vs 100	0.0786	0.0393	7.88	3.94	3.190

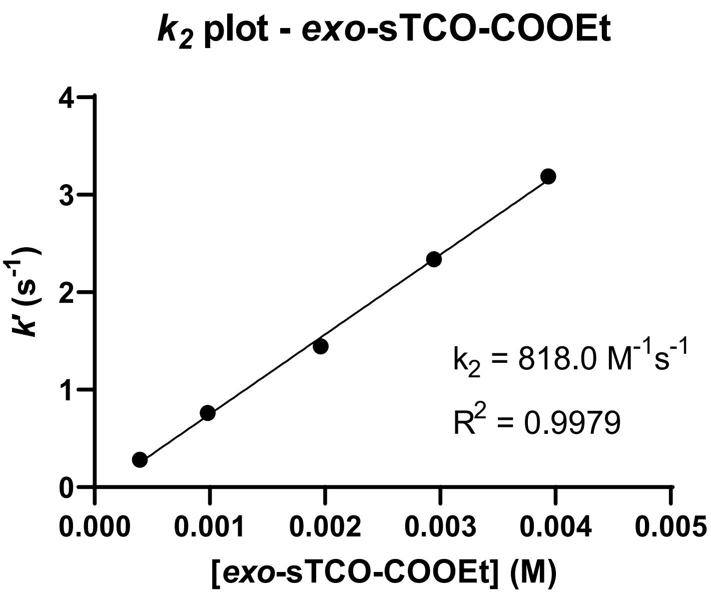

Best-fit values

Slope	818.0
Y-intercept	-0.06933
X-intercept	8.476e-005
1/slope	0.001222

Std. Error

Slope	21.58
Y-intercept	0.05212

Goodness of Fit

R squared	0.9979
Sy.x	0.06200

Equation Y = 818.0*X - 0.06933

B.5. k_2 plot *exo*-BCN-CH_2OH 8

Stoichiometry	Initial concentration *o*-quinone [mM]	Final concentration *o*-quinone [mM]	Initial concentration probe [mM]	Final concentration probe [mM]	k' (s^{-1})
1 vs 10	0.0864	0.0432	0.865	0.433	0.6111
1 vs 25	0.0864	0.0432	2.16	1.08	1.696
1 vs 50	0.0864	0.0432	4.33	2.16	3.591
1 vs 75	0.0864	0.0432	6.49	3.25	5.354
1 vs 100	0.0864	0.0432	8.65	4.33	7.164

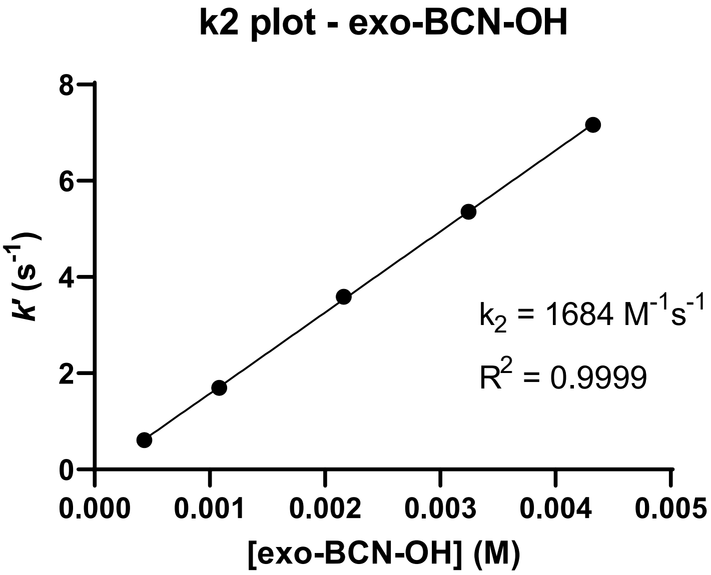

Best-fit values

Slope	1684
Y-intercept	-0.1058
X-intercept	6.280e-005
1/slope	0.0005938

Std. Error

Slope	11.12
Y-intercept	0.02953

Goodness of Fit

R squared	0.9999
Sy.x	0.03511

Equation Y = 1684*X - 0.1058

B.6. k_2 determination DBCO-acid potassium salt **10**

Stoichiometry	Initial conc. *o*-quinone [mM]	Final conc. *o*-quinone [mM]	Initial conc. probe [mM]	Final conc. probe [mM]	k' (s^{-1})	k_2 ($M^{-1}s^{-1}$)
1 vs 10	0.786	0.393	7.729	3.864	0.0006418	0.16607

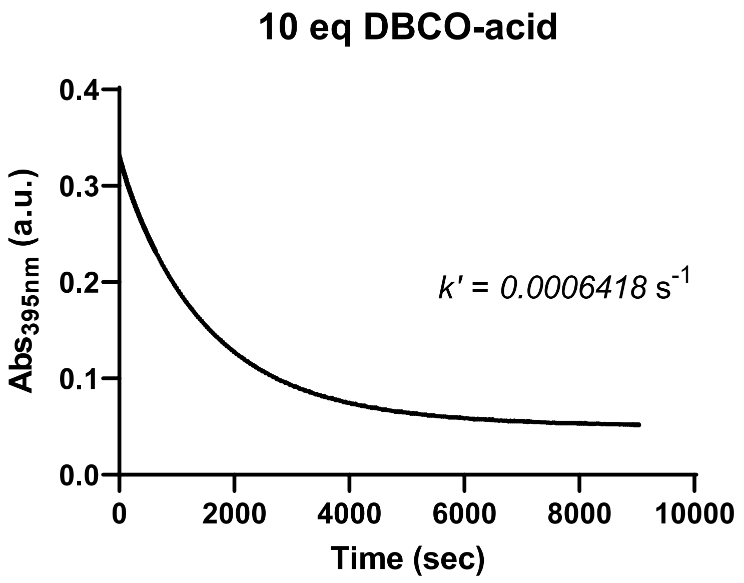

***Appendix C*: Eyring plot experiments**

C.1. Eyring plot *endo*-sTCO-CH_2OH **2**

Stoichiometry	Initial concentration *o*-quinone [mM]	Final concentration *o*-quinone [mM]	Initial concentration probe [mM]	Final concentration probe [mM]
1 *vs* 10	0.0968	0.0484	0.966	0.483

Temperature (°C)	Temperature (K)	1/T (K^{-1})	*k'* (s^{-1})	k_2 ($M^{-1}s^{-1}$)	k_2/T	ln(k_2/T)
5	278.15	0.003595	1.310	2713.34	9.754951	2.277775
13	286.15	0.003495	1.415	2930.82	10.242255	2.326522
21	294.15	0.003400	1.516	3140.02	10.674886	2.367894
29	302.15	0.003310	1.617	3349.21	11.084607	2.405557
37	310.15	0.003224	1.670	3458.99	11.152637	2.411676

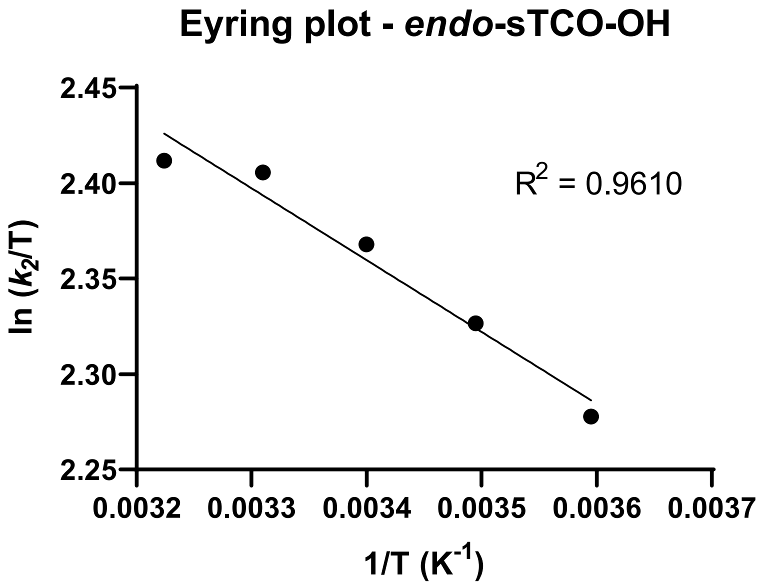

Best-fit values
- Slope -376.2
- Y-intercept 3.639
- X-intercept 0.009672
- 1/slope -0.002658

Std. Error
- Slope 43.74
- Y-intercept 0.1490

Goodness of Fit
- R squared 0.9610
- Sy.x 0.01283

Equation Y = -376.2*X + 3.639

C.2. Eyring plot *exo*-sTCO-CH_2OH **3**

Stoichiometry	Initial concentration *o*-quinone [mM]	Final concentration *o*-quinone [mM]	Initial concentration probe [mM]	Final concentration probe [mM]
1 *vs* 10	0.0871	0.0435	0.874	0.437

Temperature (°C)	Temperature (K)	1/T (K^{-1})	k' (s^{-1})	k_2 ($M^{-1}s^{-1}$)	k_2/T	ln(k_2/T)
5	278.15	0.003595	1.491	3413.314	12.271486	2.507278
13	286.15	0.003495	1.595	3651.399	12.760436	2.546349
21	294.15	0.003400	1.685	3857.434	13.113832	2.573668
29	302.15	0.003310	-	-	-	-
37	310.15	0.003224	-	-	-	-

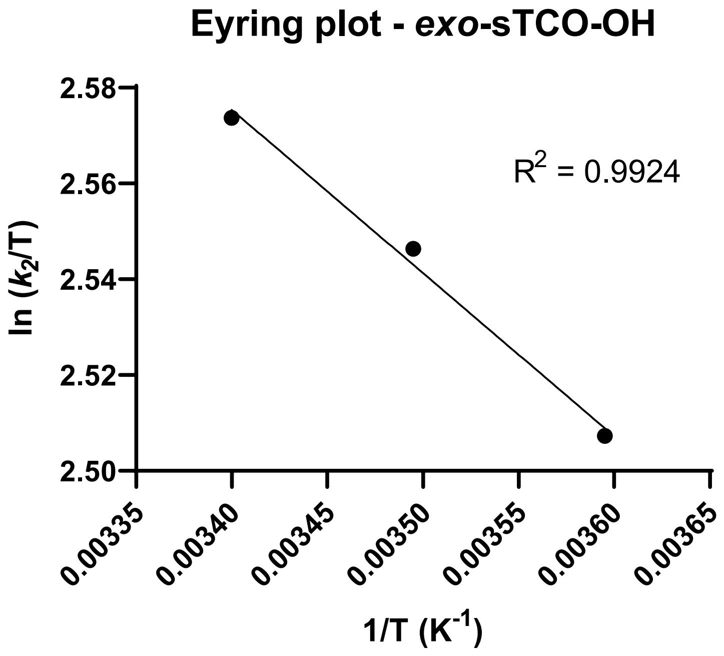

Best-fit values
- Slope -340.9
- Y-intercept 3.734
- X-intercept 0.01095
- 1/slope -0.002933

Std. Error
- Slope 29.75
- Y-intercept 0.1040

Goodness of Fit
- R squared 0.9924
- Sy.x 0.004102

Equation Y = -340.9*X + 3.734

C.3. Eyring plot *endo*-sTCO-C(O)OEt **4**

Stoichiometry	Initial concentration *o*-quinone [mM]	Final concentration *o*-quinone [mM]	Initial concentration probe [mM]	Final concentration probe [mM]
1 *vs* 10	0.0743	0.0371	0.741	0.371

Temperature (°C)	Temperature (K)	1/T (K^{-1})	k' (s^{-1})	k_2 ($M^{-1}s^{-1}$)	k_2/T	ln(k_2/T)
5	278.15	0.003595	0.6049	1632.138	5.867833	1.769485
13	286.15	0.003495	0.6667	1798.886	6.286515	1.838407
21	294.15	0.003400	0.7184	1938.383	6.589777	1.885520
29	302.15	0.003310	0.7647	2063.309	6.828758	1.921143
37	310.15	0.003224	-	-	-	-

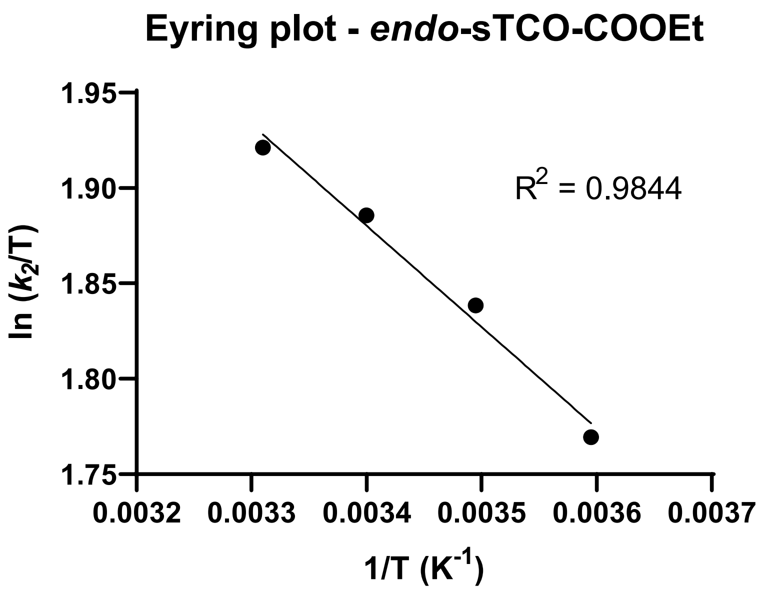

Best-fit values
  Slope -530.1
  Y-intercept 3.682
  X-intercept 0.006947
  1/slope -0.001887
Std. Error
  Slope 47.23
  Y-intercept 0.1630
Goodness of Fit
  R squared 0.9844
  Sy.x 0.01004
Equation Y = -530.1*X + 3.682

C.4. Eyring plot *exo*-sTCO-C(O)OEt **5**

Stoichiometry	Initial concentration *o*-quinone [mM]	Final concentration *o*-quinone [mM]	Initial concentration probe [mM]	Final concentration probe [mM]
1 *vs* 10	0.0786	0.0393	0.788	0.394

Temperature (°C)	Temperature (K)	1/T (K^{-1})	k' (s^{-1})	k_2 ($M^{-1}s^{-1}$)	k_2/T	ln(k_2/T)
5	278.15	0.003595	0.2335	592.968	2.131828	0.756980
13	286.15	0.003495	0.2539	644.773	2.253270	0.812383
21	294.15	0.003400	0.2771	703.689	2.392280	0.872247
29	302.15	0.003310	0.2998	761.335	2.519726	0.924150
37	310.15	0.003224	0.3200	812.633	2.620128	0.963223

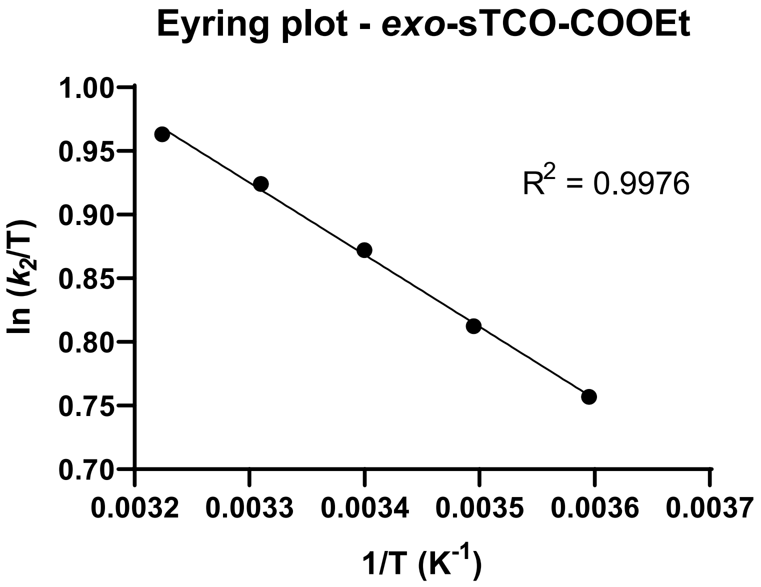

Best-fit values
  Slope -566.1
  Y-intercept 2.793
  X-intercept 0.004934
  1/slope -0.001766
Std. Error
  Slope 16.11
  Y-intercept 0.05490
Goodness of Fit
  R squared 0.9976
  Sy.x 0.004726
Equation Y = -566.1*X + 2.793

C.5. Eyring plot TCO-OH **6**

Stoichiometry	Initial concentration *o*-quinone [mM]	Final concentration *o*-quinone [mM]	Initial concentration probe [mM]	Final concentration probe [mM]
1 *vs* 10	0.0968	0.0484	0.967	0.483

Temperature (°C)	Temperature (K)	1/T (K^{-1})	k' (s^{-1})	k_2 ($M^{-1}s^{-1}$)	k_2/T	ln(k_2/T)
5	278.15	0.003595	0.004761	9.850	0.035412	-3.340717
13	286.15	0.003495	0.004975	10.292	0.035969	-3.325105
21	294.15	0.003400	0.005297	10.959	0.037255	-3.289963
29	302.15	0.003310	0.005570	11.523	0.038138	-3.266542
37	310.15	0.003224	-	-	-	-

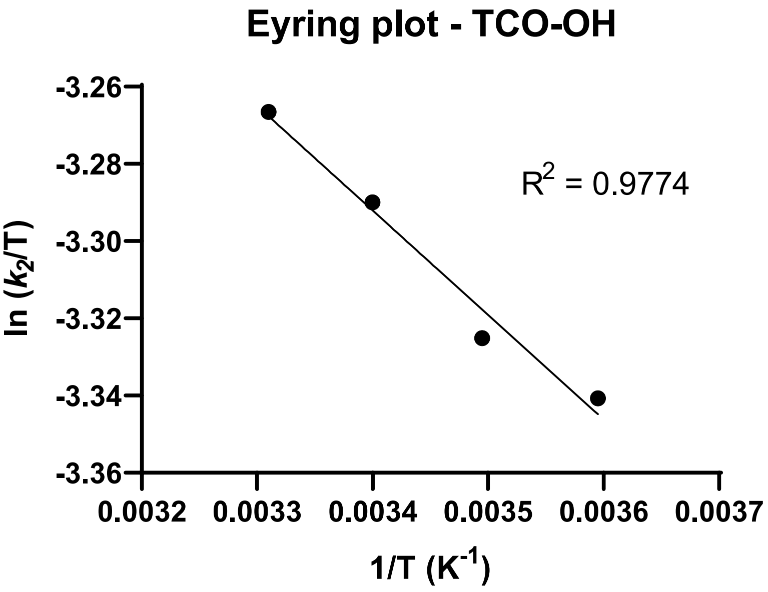

Best-fit values
  Slope -270.7
  Y-intercept -2.372
  X-intercept -0.008763
  1/slope -0.003695
Std. Error
  Slope 29.09
  Y-intercept 0.1004
Goodness of Fit
  R squared 0.9774
  Sy.x 0.006181
Equation Y = -270.7*X - 2.372

C.6. Eyring plot *exo*-BCN-CH_2OH **8**

Stoichiometry	Initial concentration *o*-quinone [mM]	Final concentration *o*-quinone [mM]	Initial concentration probe [mM]	Final concentration probe [mM]
1 *vs* 10	0.0864	0.0432	0.865	0.433

Temperature (°C)	Temperature (K)	1/T (K^{-1})	k' (s^{-1})	k_2 ($M^{-1}s^{-1}$)	k_2/T	ln(k_2/T)
5	278.15	0.003595	0.4711	1088.756	3.914275	1.364630
13	286.15	0.003495	0.5033	1163.173	4.064906	1.402391
21	294.15	0.003400	0.5889	1361.002	4.626899	1.531887
29	302.15	0.003310	0.665	1536.876	5.086468	1.626584
37	310.15	0.003224	0.7024	1623.311	5.233955	1.655167

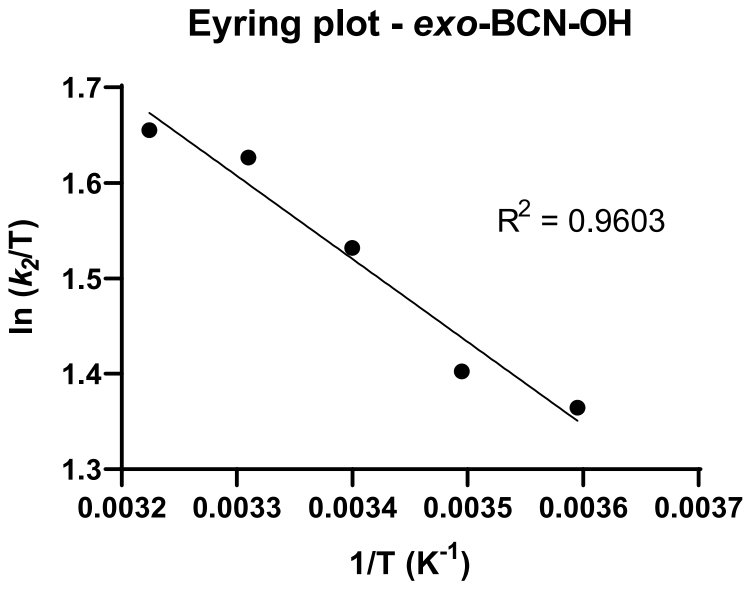

Best-fit values
Slope -869.2
Y-intercept 4.476
X-intercept 0.005149
1/slope -0.001151
Std. Error
Slope 102.1
Y-intercept 0.3478
Goodness of Fit
R squared 0.9603
Sy.x 0.02994
Equation Y = -869.2*X + 4.476

C.7. Kinetics of axial TCO-OH (**6-ax**) *versus* equatorial TCO-OH (**6-eq**)

C.7a. k' data for k_2 determination TCO-OH **6-ax** and **6-eq**

Stoichiometry	Initial conc. *o*-quinone [mM]	Final conc. *o*-quinone [mM]	Initial conc. probe [mM]	Final conc. probe [mM]	k' (s^{-1})	k_2 ($M^{-1}s^{-1}$)
1 *vs* 50 (**6-ax**)	0.0864	0.0432	4.10	2.05	0.07204	34.85
1 *vs* 50 (**6-eq**)	0.0864	0.0432	4.13	2.07	0.00576	2.81

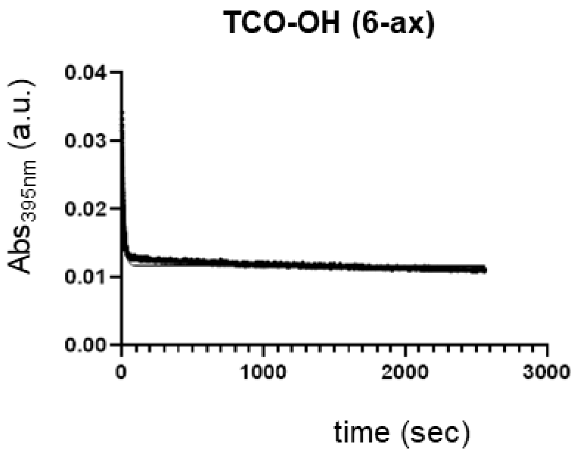

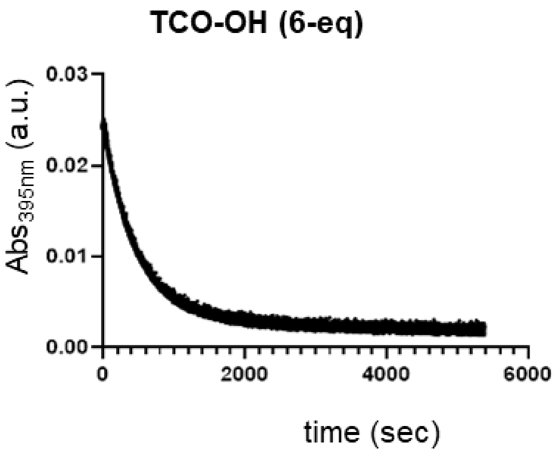

Eyring plot

Stoichiometry	Initial concentration *o*-quinone [mM]	Final concentration *o*-quinone [mM]	Initial concentration probe [mM]	Final concentration probe [mM]
1 *vs* 50 (**6-ax**)	0.0864	0.0432	4.10	2.05

TCO-OH (**6-ax**) Temperature (°C)	T (K)	1/T (K^{-1})	k_2 ($M^{-1}s^{-1}$)	k_2/T	ln(k_2/T)
5	278.15	0.003595	27.07	0.097	-2.330
13	286.15	0.003495	32.49	0.114	-2.176
21	294.15	0.003400	35.66	0.121	-2.110
29	302.15	0.003310	39.97	0.132	-2.023

***Appendix D*: Kinetic studies in MeOH**

D.1. *k′* data for k_2 determination for *endo*-sTCO-C(O)OEt (**4**) and *exo*-sTCO-C(O)OEt (**5**) in MeOH

endo-sTCO-C(O)OEt (**4**)

Stoichiometry	Initial concentration *o*-quinone [mM]	Final concentration *o*-quinone [mM]	Initial concentration probe [mM]	Final concentration probe [mM]	*k′* (s^{-1})
1 vs 2	0.0396	0.0198	0.0793	0.0397	0.001869
1 vs 5	0.0396	0.0198	0.1981	0.0991	0.004735
1 vs 10	0.0396	0.0198	0.3963	0.1982	0.012430
1 vs 25	0.0396	0.0198	0.9907	0.4954	0.042000
1 vs 50	0.0396	0.0198	1.9814	0.9907	0.076133

exo-sTCO-C(O)OEt (**5**)

Stoichiometry	Initial concentration *o*-quinone [mM]	Final concentration *o*-quinone [mM]	Initial concentration probe [mM]	Final concentration probe [mM]	*k′* (s^{-1})
1 vs 2	0.0396	0.0198	0.0786	0.0393	0.000693
1 vs 5	0.0396	0.0198	0.1966	0.0983	0.001909
1 vs 10	0.0396	0.0198	0.3932	0.1966	0.004677
1 vs 25	0.0396	0.0198	0.9830	0.4915	0.009459
1 vs 50	0.0396	0.0198	1.9660	0.9830	0.018427

k_2-plot *endo*-sTCO-C(O)OEt (**4**)

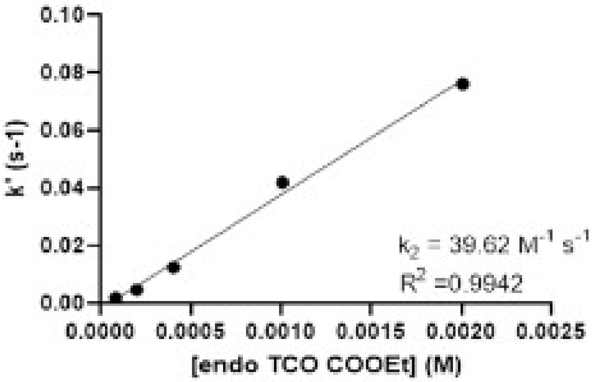

k_2-plot *exo*-sTCO-C(O)OEt (**5**)

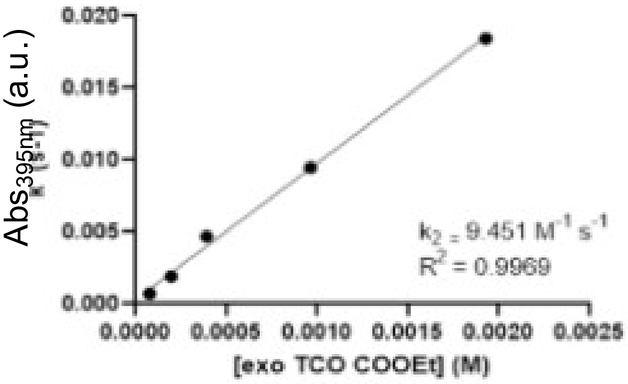

D.2. Eyring analysis of *endo*-sTCO-CH_2OH (**2**) and *exo*-sTCO-CH_2OH (**3**) in MeOH

endo-sTCO-CH_2OH (**2**)

T (°C)	T (K)	1/T (K^{-1})	k_2 ($M^{-1}s^{-1}$)	k_2/T	ln(k_2/T)
5	278.15	0.003595	46.09	0.165690	-1.79764
13	286.15	0.003495	53.11	0.185617	-1.68407
21	294.15	0.003400	56.46	0.191943	-1.65056
29	302.15	0.003310	59.43	0.196679	-1.62618
37	310.15	0.003224	64.27	0.207222	-1.57396

exo-sTCO-CH_2OH (**3**)

T (°C)	T (K)	1/T (K^{-1})	k_2 ($M^{-1}s^{-1}$)	k_2/T	ln(k_2/T)
5	278.15	0.003595	26.33	0.094654	-2.35753
13	286.15	0.003495	29.31	0.102411	-2.27876
21	294.15	0.003400	31.80	0.114082	-2.22478
29	302.15	0.003310	34.47	0.114082	-2.17083
37	310.15	0.003224	36.26	0.116913	-2.14633

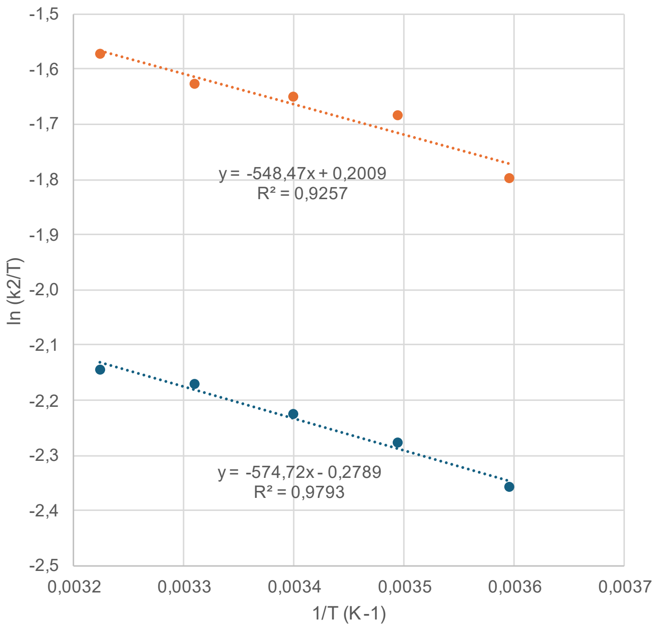

Eyring plot *endo*-sTCO-CH_2OH (**2**; orange) and *exo*-sTCO-CH_2OH (**3**; blue).

Appendix E: **Fluorescence spectroscopy kinetic studies**

E.1. Results SPAAC fluorescence spectroscopy

Stoichiometry	Initial concentration 3-azido-7-hydroxycoumarin [µM]	Final concentration 3-azido-7-hydroxycoumarin [µM]
1 *vs* 1000	19.7	9.84

probe	Run	Initial concentration probe [mM]	Final concentration probe [mM]	*k'* (s^{-1})	k_2 ($M^{-1}s^{-1}$)	Average k_2 (± S.D.)
endo-BCN-CH_2OH **7**	1	20.1	10.05	0.009203	0.915546	0.900425 $M^{-1}s^{-1}$ (± 0.084783)
	2	20.1	10.05	0.008133	0.809099	
	3	20.1	10.05	0.009817	0.976629	
exo-BCN-CH_2OH **8**	1	20.8	10.38	0.009203	0.886202	0.882639 $M^{-1}s^{-1}$ (± 0.058196)
	2	20.8	10.38	0.009751	0.938971	
	3	20.8	10.38	0.008544	0.822743	

Appendix F: XRD of *endo*-BCN-OH

F.1. XRD Crystal structure of *endo*-BCN-OH **7**:

The XRD quality crystals were grown from a hot Et_2O solution.

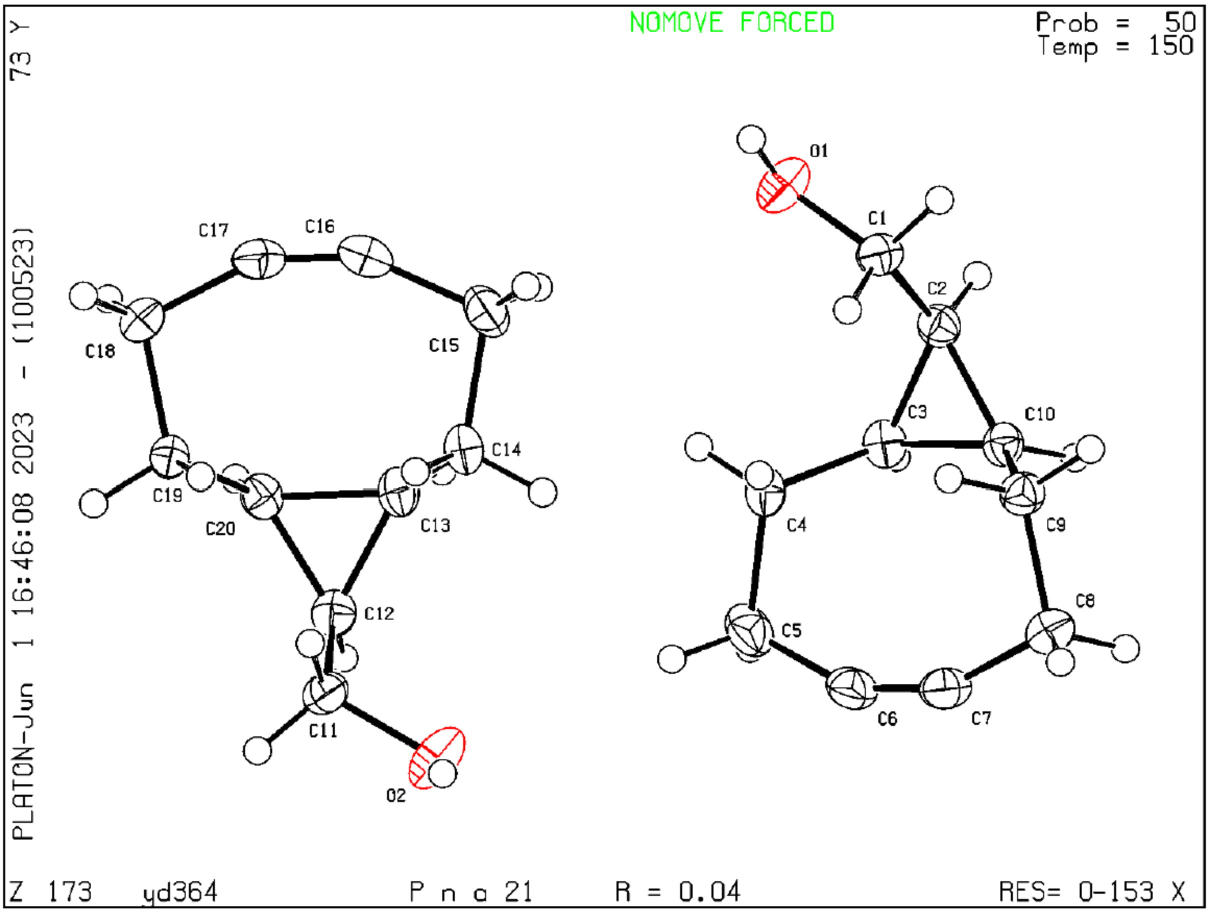

Figure S1: Molecular structure of ***endo*-BCN-CH_2OH 7** according to X-ray structure determination. ORTEP depicted with thermal ellipsoids drawn at 50% probability level.

Table S4. Crystal data and structure refinement for *endo*-BCN-CH_2OH **7**.

Identification code	yd364	
Empirical formula	C10 H14 O	
Formula weight	150.21	
Temperature	150.01(10) K	
Wavelength	1.54184 Å	
Crystal system	Orthorhombic	
Space group	$Pna2_1$	
Unit cell dimensions	a = 9.34904(9) Å	α= 90°
	b = 8.21004(7) Å	β= 90°
	c = 22.1590(2) Å	γ = 90°
Volume	1700.84(3) $Å^3$	
Z	8	
Density (calculated)	1.173 Mg/m^3	
Absorption coefficient	0.570 mm^{-1}	
F(000)	656	
Crystal size	0.265 x 0.190 x 0.156 mm^3	
Theta range for data collection	3.990 to 72.922°	
Index ranges	-11<=h<=11, -10<=k<=10, -27<=l<=27	
Reflections collected	27802	
Independent reflections	3389 [R(int) = 0.0312]	
Completeness to theta = 67.684°	100.0 %	
Absorption correction	Semi-empirical from equivalents	
Max. and min. transmission	1.00000 and 0.89004	
Refinement method	Full-matrix least-squares on F^2	
Data / restraints / parameters	3389 / 1 / 208	
Goodness-of-fit on F^2	1.073	
Final R indices [I>2sigma(I)]	R1 = 0.0380, wR2 = 0.1009	
R indices (all data)	R1 = 0.0383, wR2 = 0.1013	
Absolute structure parameter	0.4(3)	
Extinction coefficient	n/a	
Largest diff. peak and hole	0.318 and -0.147 e.$Å^{-3}$	

Table S5. Atomic coordinates (x 10^4) and equivalent isotropic displacement parameters ($Å^2$x 10^3) for *endo*-BCN-OH **7**. U(eq) is defined as one third of the trace of the orthogonalized U^{ij} tensor.

	x	y	z	U(eq)
O(1)	4573(2)	11818(2)	4744(1)	35(1)
C(1)	4849(2)	11441(2)	4124(1)	25(1)
C(2)	3556(2)	10615(2)	3868(1)	24(1)
C(3)	3303(2)	8812(2)	3969(1)	24(1)
C(4)	4259(2)	7791(3)	4366(1)	26(1)
C(5)	4119(3)	5936(3)	4234(1)	34(1)
C(6)	4471(3)	5745(2)	3594(1)	33(1)
C(7)	4751(2)	6235(3)	3101(1)	32(1)
C(8)	5020(3)	7504(3)	2647(1)	32(1)
C(9)	5063(2)	9126(2)	3006(1)	25(1)
C(10)	3678(2)	9432(2)	3343(1)	22(1)
O(2)	6906(2)	3254(2)	5231(1)	39(1)
C(11)	7119(2)	3580(2)	5860(1)	27(1)
C(12)	5826(2)	4427(3)	6101(1)	25(1)
C(13)	5609(2)	6240(3)	6008(1)	24(1)
C(14)	6632(2)	7252(3)	5636(1)	26(1)
C(15)	6506(3)	9097(3)	5768(1)	34(1)
C(16)	6786(3)	9263(2)	6419(1)	33(1)
C(17)	7016(2)	8749(2)	6910(1)	31(1)
C(18)	7236(3)	7453(2)	7358(1)	31(1)
C(19)	7285(2)	5850(2)	6993(1)	25(1)
C(20)	5921(2)	5578(2)	6635(1)	23(1)

Table S6. Bond lengths [Å] for *endo*-BCN-CH_2OH **7**.

O(1)-C(1)	1.430(2)
O(1)-H(1)	0.90(4)
C(1)-C(2)	1.497(3)
C(1)-H(1A)	0.9900
C(1)-H(1B)	0.9900
C(2)-C(3)	1.516(3)
C(2)-C(10)	1.520(3)
C(2)-H(2)	1.0000
C(3)-C(4)	1.508(3)
C(3)-C(10)	1.520(3)
C(3)-H(3)	1.0000
C(4)-C(5)	1.557(3)
C(4)-H(4A)	0.9900
C(4)-H(4B)	0.9900
C(5)-C(6)	1.464(3)
C(5)-H(5A)	0.9900
C(5)-H(5B)	0.9900
C(6)-C(7)	1.193(3)
C(7)-C(8)	1.470(3)
C(8)-C(9)	1.552(3)
C(8)-H(8A)	0.9900
C(8)-H(8B)	0.9900
C(9)-C(10)	1.516(3)
C(9)-H(9A)	0.9900
C(9)-H(9B)	0.9900
C(10)-H(10)	1.0000
O(2)-C(11)	1.433(2)
O(2)-H(2A)	0.76(4)
C(11)-C(12)	1.494(3)
C(11)-H(11A)	0.9900
C(11)-H(11B)	0.9900
C(12)-C(13)	1.516(3)
C(12)-C(20)	1.518(3)
C(12)-H(12)	1.0000
C(13)-C(14)	1.512(3)
C(13)-C(20)	1.521(3)

C(13)-H(13)	1.0000
C(14)-C(15)	1.547(3)
C(14)-H(14A)	0.9900
C(14)-H(14B)	0.9900
C(15)-C(16)	1.472(3)
C(15)-H(15A)	0.9900
C(15)-H(15B)	0.9900
C(16)-C(17)	1.187(4)
C(17)-C(18)	1.470(3)
C(18)-C(19)	1.546(3)
C(18)-H(18A)	0.9900
C(18)-H(18B)	0.9900
C(19)-C(20)	1.518(3)
C(19)-H(19A)	0.9900
C(19)-H(19B)	0.9900
C(20)-H(20)	1.0000

Table S7. Bond angles [°] for *endo*-BCN-CH_2OH **7**.

C(1)-O(1)-H(1)	108(2)
O(1)-C(1)-C(2)	108.46(16)
O(1)-C(1)-H(1A)	110.0
C(2)-C(1)-H(1A)	110.0
O(1)-C(1)-H(1B)	110.0
C(2)-C(1)-H(1B)	110.0
H(1A)-C(1)-H(1B)	108.4
C(1)-C(2)-C(3)	120.83(17)
C(1)-C(2)-C(10)	121.29(17)
C(3)-C(2)-C(10)	60.09(12)
C(1)-C(2)-H(2)	114.6
C(3)-C(2)-H(2)	114.6
C(10)-C(2)-H(2)	114.6
C(4)-C(3)-C(2)	122.45(17)
C(4)-C(3)-C(10)	125.56(17)
C(2)-C(3)-C(10)	60.10(12)
C(4)-C(3)-H(3)	112.9
C(2)-C(3)-H(3)	112.9
C(10)-C(3)-H(3)	112.9
C(3)-C(4)-C(5)	112.64(17)
C(3)-C(4)-H(4A)	109.1
C(5)-C(4)-H(4A)	109.1
C(3)-C(4)-H(4B)	109.1
C(5)-C(4)-H(4B)	109.1
H(4A)-C(4)-H(4B)	107.8
C(6)-C(5)-C(4)	105.54(17)
C(6)-C(5)-H(5A)	110.6
C(4)-C(5)-H(5A)	110.6
C(6)-C(5)-H(5B)	110.6
C(4)-C(5)-H(5B)	110.6
H(5A)-C(5)-H(5B)	108.8
C(7)-C(6)-C(5)	154.2(2)
C(6)-C(7)-C(8)	154.5(2)
C(7)-C(8)-C(9)	105.23(18)
C(7)-C(8)-H(8A)	110.7
C(9)-C(8)-H(8A)	110.7

C(7)-C(8)-H(8B)	110.7
C(9)-C(8)-H(8B)	110.7
H(8A)-C(8)-H(8B)	108.8
C(10)-C(9)-C(8)	111.86(17)
C(10)-C(9)-H(9A)	109.2
C(8)-C(9)-H(9A)	109.2
C(10)-C(9)-H(9B)	109.2
C(8)-C(9)-H(9B)	109.2
H(9A)-C(9)-H(9B)	107.9
C(9)-C(10)-C(3)	126.29(17)
C(9)-C(10)-C(2)	123.16(16)
C(3)-C(10)-C(2)	59.81(12)
C(9)-C(10)-H(10)	112.6
C(3)-C(10)-H(10)	112.6
C(2)-C(10)-H(10)	112.6
C(11)-O(2)-H(2A)	106(3)
O(2)-C(11)-C(12)	108.83(17)
O(2)-C(11)-H(11A)	109.9
C(12)-C(11)-H(11A)	109.9
O(2)-C(11)-H(11B)	109.9
C(12)-C(11)-H(11B)	109.9
H(11A)-C(11)-H(11B)	108.3
C(11)-C(12)-C(13)	121.13(17)
C(11)-C(12)-C(20)	121.50(17)
C(13)-C(12)-C(20)	60.16(13)
C(11)-C(12)-H(12)	114.5
C(13)-C(12)-H(12)	114.5
C(20)-C(12)-H(12)	114.5
C(14)-C(13)-C(12)	121.93(18)
C(14)-C(13)-C(20)	124.99(17)
C(12)-C(13)-C(20)	59.96(13)
C(14)-C(13)-H(13)	113.3
C(12)-C(13)-H(13)	113.3
C(20)-C(13)-H(13)	113.3
C(13)-C(14)-C(15)	112.76(18)
C(13)-C(14)-H(14A)	109.0
C(15)-C(14)-H(14A)	109.0
C(13)-C(14)-H(14B)	109.0

C(15)-C(14)-H(14B)	109.0
H(14A)-C(14)-H(14B)	107.8
C(16)-C(15)-C(14)	105.20(17)
C(16)-C(15)-H(15A)	110.7
C(14)-C(15)-H(15A)	110.7
C(16)-C(15)-H(15B)	110.7
C(14)-C(15)-H(15B)	110.7
H(15A)-C(15)-H(15B)	108.8
C(17)-C(16)-C(15)	153.9(2)
C(16)-C(17)-C(18)	154.4(2)
C(17)-C(18)-C(19)	105.46(18)
C(17)-C(18)-H(18A)	110.6
C(19)-C(18)-H(18A)	110.6
C(17)-C(18)-H(18B)	110.6
C(19)-C(18)-H(18B)	110.6
H(18A)-C(18)-H(18B)	108.8
C(20)-C(19)-C(18)	111.93(17)
C(20)-C(19)-H(19A)	109.2
C(18)-C(19)-H(19A)	109.2
C(20)-C(19)-H(19B)	109.2
C(18)-C(19)-H(19B)	109.2
H(19A)-C(19)-H(19B)	107.9
C(12)-C(20)-C(19)	123.21(17)
C(12)-C(20)-C(13)	59.88(13)
C(19)-C(20)-C(13)	125.85(17)
C(12)-C(20)-H(20)	112.7
C(19)-C(20)-H(20)	112.7
C(13)-C(20)-H(20)	112.7

Table S8. Anisotropic displacement parameters ($Å^2 x 10^3$) for *endo*-BCN-CH_2OH **7**. The anisotropic displacement factor exponent takes the form: $-2\pi^2[h^2 a^{*2}U^{11} + ... + 2 h k a^* b^* U^{12}]$

	U^{11}	U^{22}	U^{33}	U^{23}	U^{13}	U^{12}
O(1)	33(1)	45(1)	26(1)	-8(1)	1(1)	-9(1)
C(1)	27(1)	23(1)	25(1)	-2(1)	2(1)	-1(1)
C(2)	23(1)	24(1)	24(1)	0(1)	-1(1)	3(1)
C(3)	18(1)	28(1)	25(1)	3(1)	0(1)	-2(1)
C(4)	26(1)	29(1)	21(1)	4(1)	-1(1)	-1(1)
C(5)	38(1)	27(1)	38(1)	10(1)	-6(1)	-7(1)
C(6)	38(1)	20(1)	40(1)	0(1)	-8(1)	-3(1)
C(7)	38(1)	24(1)	35(1)	-7(1)	-4(1)	1(1)
C(8)	39(1)	29(1)	27(1)	-5(1)	0(1)	1(1)
C(9)	26(1)	23(1)	25(1)	-1(1)	1(1)	-2(1)
C(10)	20(1)	24(1)	22(1)	1(1)	-2(1)	-1(1)
O(2)	32(1)	59(1)	26(1)	-12(1)	2(1)	-10(1)
C(11)	31(1)	25(1)	24(1)	-4(1)	-1(1)	-2(1)
C(12)	23(1)	28(1)	26(1)	2(1)	-2(1)	-6(1)
C(13)	19(1)	29(1)	26(1)	3(1)	-4(1)	1(1)
C(14)	27(1)	29(1)	22(1)	6(1)	-1(1)	1(1)
C(15)	38(1)	28(1)	36(1)	9(1)	-5(1)	4(1)
C(16)	37(1)	20(1)	41(1)	0(1)	1(1)	3(1)
C(17)	35(1)	24(1)	33(1)	-6(1)	3(1)	2(1)
C(18)	43(1)	29(1)	21(1)	-4(1)	-1(1)	3(1)
C(19)	30(1)	25(1)	20(1)	-1(1)	-3(1)	4(1)
C(20)	22(1)	24(1)	24(1)	3(1)	3(1)	0(1)

Table S9. Hydrogen coordinates (x 10^4) and isotropic displacement parameters ($Å^2$x 10^3) for *endo*-BCN-CH_2OH **7**.

	x	y	z	U(eq)
H(1)	5330(40)	12370(40)	4888(17)	64(10)
H(1A)	5691	10716	4092	30
H(1B)	5052	12453	3897	30
H(2)	2670	11296	3866	29
H(3)	2267	8534	4014	29
H(4A)	4014	7996	4794	31
H(4B)	5265	8128	4304	31
H(5A)	4791	5304	4488	41
H(5B)	3133	5555	4316	41
H(8A)	4246	7524	2343	38
H(8B)	5942	7310	2440	38
H(9A)	5865	9092	3298	30
H(9B)	5241	10038	2723	30
H(10)	2830	9475	3068	27
H(2A)	7650(40)	3200(40)	5090(15)	49(9)
H(11A)	7975	4274	5915	32
H(11B)	7274	2546	6080	32
H(12)	4928	3767	6085	30
H(13)	4584	6548	5949	29
H(14A)	6440	7059	5203	31
H(14B)	7624	6894	5721	31
H(15A)	7218	9719	5531	41
H(15B)	5537	9500	5667	41
H(18A)	6440	7432	7653	37
H(18B)	8144	7622	7579	37
H(19A)	8108	5883	6712	30
H(19B)	7430	4925	7273	30
H(20)	5050	5540	6897	28

Table S10. Torsion angles [°] for *endo*-BCN-CH_2OH **7**.

O(1)-C(1)-C(2)-C(3)	-81.4(2)
O(1)-C(1)-C(2)-C(10)	-152.94(18)
C(1)-C(2)-C(3)-C(4)	4.7(3)
C(10)-C(2)-C(3)-C(4)	115.4(2)
C(1)-C(2)-C(3)-C(10)	-110.8(2)
C(2)-C(3)-C(4)-C(5)	-160.52(17)
C(10)-C(3)-C(4)-C(5)	-86.3(2)
C(3)-C(4)-C(5)-C(6)	57.7(2)
C(4)-C(5)-C(6)-C(7)	-6.5(6)
C(5)-C(6)-C(7)-C(8)	0.0(10)
C(6)-C(7)-C(8)-C(9)	7.6(6)
C(7)-C(8)-C(9)-C(10)	-58.8(2)
C(8)-C(9)-C(10)-C(3)	86.5(2)
C(8)-C(9)-C(10)-C(2)	161.08(18)
C(4)-C(3)-C(10)-C(9)	0.5(3)
C(2)-C(3)-C(10)-C(9)	111.0(2)
C(4)-C(3)-C(10)-C(2)	-110.5(2)
C(1)-C(2)-C(10)-C(9)	-5.9(3)
C(3)-C(2)-C(10)-C(9)	-116.0(2)
C(1)-C(2)-C(10)-C(3)	110.0(2)
O(2)-C(11)-C(12)-C(13)	-80.4(2)
O(2)-C(11)-C(12)-C(20)	-152.27(18)
C(11)-C(12)-C(13)-C(14)	3.9(3)
C(20)-C(12)-C(13)-C(14)	114.8(2)
C(11)-C(12)-C(13)-C(20)	-110.9(2)
C(12)-C(13)-C(14)-C(15)	-161.21(18)
C(20)-C(13)-C(14)-C(15)	-87.7(2)
C(13)-C(14)-C(15)-C(16)	57.9(2)
C(14)-C(15)-C(16)-C(17)	-4.5(6)
C(15)-C(16)-C(17)-C(18)	-1.3(10)
C(16)-C(17)-C(18)-C(19)	7.0(6)
C(17)-C(18)-C(19)-C(20)	-58.8(2)
C(11)-C(12)-C(20)-C(19)	-5.1(3)
C(13)-C(12)-C(20)-C(19)	-115.4(2)
C(11)-C(12)-C(20)-C(13)	110.3(2)
C(18)-C(19)-C(20)-C(12)	160.72(18)

C(18)-C(19)-C(20)-C(13)	86.2(2)
C(14)-C(13)-C(20)-C(12)	-109.9(2)
C(14)-C(13)-C(20)-C(19)	1.3(3)
C(12)-C(13)-C(20)-C(19)	111.2(2)

Table S11. Hydrogen bonds for *endo*-BCN-CH_2OH **7** [Å and °].

D-H...A	d(D-H)	d(H...A)	d(D...A)	<(DHA)
O(1)-H(1)...O(2)#1	0.90(4)	1.81(4)	2.704(2)	173(4)
O(2)-H(2A)...O(1)#2	0.76(4)	1.96(4)	2.718(2)	177(3)

Symmetry transformations used to generate equivalent atoms:

#1 x,y+1,z   #2 x+1/2,-y+3/2,z

Appendix G: XRD of DBCO-acid

G.1 XRD Crystal structure of DBCO-acid **10**:

The XRD quality crystals were grown from a hot THF solution.

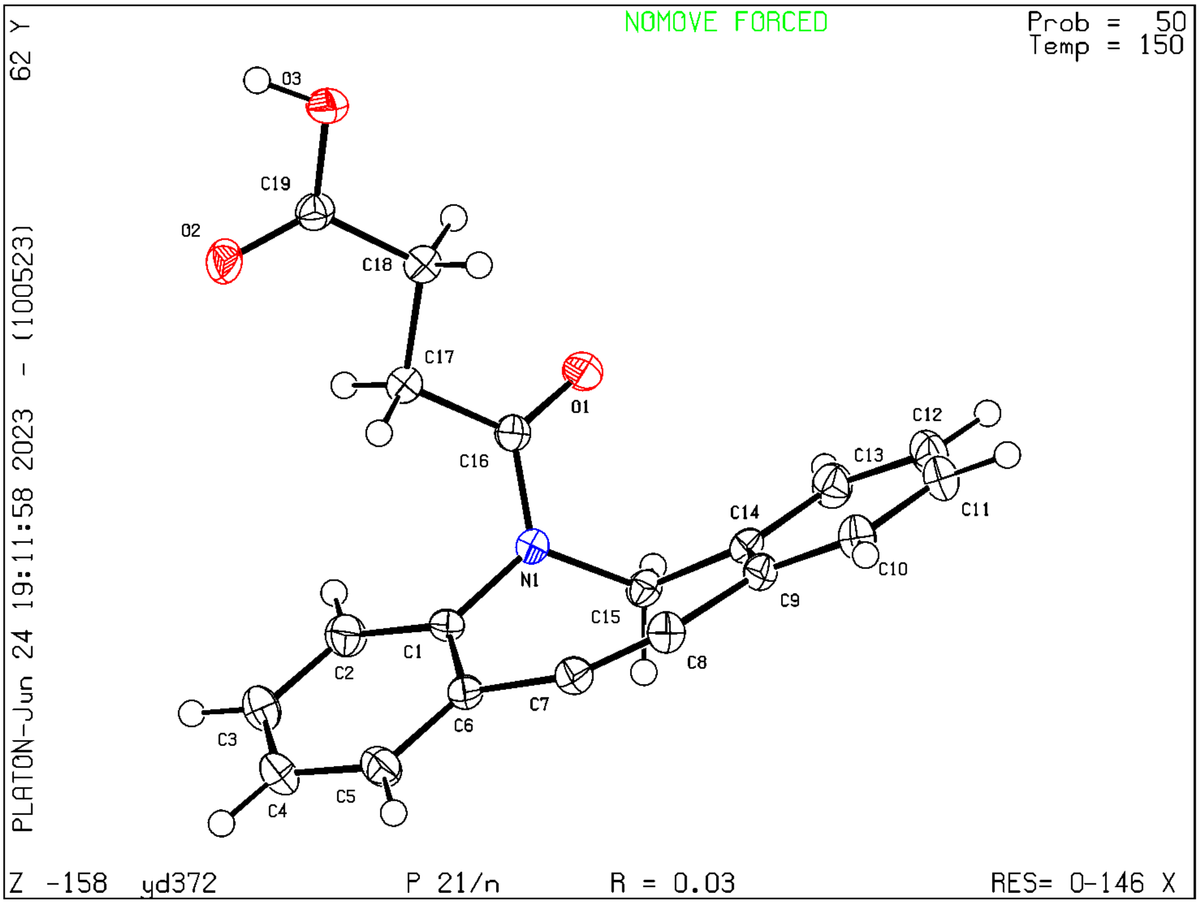

Figure S2: Molecular structure of **DBCO-acid 10** according to X-ray structure determination. ORTEP depicted with thermal ellipsoids drawn at 50% probability level.

Table S12. Crystal data and structure refinement for DBCO-acid **10**.

Identification code	yd372	
Empirical formula	C19 H15 N O3	
Formula weight	305.32	
Temperature	150.00(10) K	
Wavelength	1.54184 Å	
Crystal system	Monoclinic	
Space group	$P2_1/n$	
Unit cell dimensions	a = 5.46003(7) Å	α= 90°
	b = 16.26685(15) Å	β= 97.0485(10)°
	c = 16.91898(17) Å	γ = 90°
Volume	1491.34(3) $Å^3$	
Z	4	
Density (calculated)	1.360 Mg/m^3	
Absorption coefficient	0.752 mm^{-1}	
F(000)	640	
Crystal size	0.120 x 0.100 x 0.050 mm^3	
Theta range for data collection	3.783 to 72.957°.	
Index ranges	-5<=h<=6, -20<=k<=20, -20<=l<=20	
Reflections collected	27447	
Independent reflections	2976 [R(int) = 0.0300]	
Completeness to theta = 67.684°	100.0 %	
Absorption correction	Semi-empirical from equivalents	
Max. and min. transmission	1.00000 and 0.87623	
Refinement method	Full-matrix least-squares on F^2	
Data / restraints / parameters	2976 / 0 / 212	
Goodness-of-fit on F^2	1.063	
Final R indices [I>2sigma(I)]	R1 = 0.0339, wR2 = 0.0819	
R indices (all data)	R1 = 0.0363, wR2 = 0.0836	
Extinction coefficient	n/a	
Largest diff. peak and hole	0.214 and -0.200 e.$Å^{-3}$	

Table S13. Atomic coordinates (x 10^4) and equivalent isotropic displacement parameters ($Å^2$ x 10^3) for DBCO-acid **10**. U(eq) is defined as one third of the trace of the orthogonalized U^{ij} tensor.

	x	y	z	U(eq)
N(1)	703(2)	4966(1)	2056(1)	18(1)
O(1)	164(2)	5875(1)	3023(1)	24(1)
O(2)	7870(2)	4674(1)	4331(1)	32(1)
O(3)	8286(2)	5965(1)	4774(1)	27(1)
C(1)	1792(2)	4223(1)	1801(1)	19(1)
C(2)	1725(2)	3502(1)	2234(1)	26(1)
C(3)	2881(2)	2797(1)	2000(1)	30(1)
C(4)	4051(2)	2802(1)	1319(1)	29(1)
C(5)	4073(2)	3511(1)	866(1)	26(1)
C(6)	2970(2)	4229(1)	1102(1)	19(1)
C(7)	2843(2)	5002(1)	700(1)	20(1)
C(8)	2190(2)	5706(1)	628(1)	21(1)
C(9)	706(2)	6400(1)	783(1)	20(1)
C(10)	948(2)	7197(1)	496(1)	25(1)
C(11)	-605(2)	7810(1)	702(1)	26(1)
C(12)	-2390(2)	7634(1)	1189(1)	29(1)
C(13)	-2628(2)	6844(1)	1485(1)	26(1)
C(14)	-1099(2)	6214(1)	1288(1)	19(1)
C(15)	-1454(2)	5339(1)	1561(1)	19(1)
C(16)	1378(2)	5312(1)	2784(1)	18(1)
C(17)	3704(2)	5002(1)	3276(1)	22(1)
C(18)	4980(2)	5689(1)	3776(1)	23(1)
C(19)	7181(2)	5388(1)	4313(1)	21(1)

Table S14. Bond lengths [Å] and angles [°] for DBCO-acid **10**.

N(1)-C(16)	1.3643(14)
N(1)-C(1)	1.4362(13)
N(1)-C(15)	1.4879(13)
O(1)-C(16)	1.2275(13)
O(2)-C(19)	1.2207(14)
O(3)-C(19)	1.3176(14)
O(3)-H(3A)	0.93(2)
C(1)-C(2)	1.3848(15)
C(1)-C(6)	1.4131(15)
C(2)-C(3)	1.3895(17)
C(2)-H(2)	0.9500
C(3)-C(4)	1.3850(18)
C(3)-H(3)	0.9500
C(4)-C(5)	1.3870(17)
C(4)-H(4)	0.9500
C(5)-C(6)	1.3937(15)
C(5)-H(5)	0.9500
C(6)-C(7)	1.4274(15)
C(7)-C(8)	1.2023(16)
C(8)-C(9)	1.4319(15)
C(9)-C(10)	1.3959(15)
C(9)-C(14)	1.4136(15)
C(10)-C(11)	1.3813(16)
C(10)-H(10)	0.9500
C(11)-C(12)	1.3805(18)
C(11)-H(11)	0.9500
C(12)-C(13)	1.3914(17)
C(12)-H(12)	0.9500
C(13)-C(14)	1.3873(16)
C(13)-H(13)	0.9500
C(14)-C(15)	1.5175(15)
C(15)-H(15A)	0.9900
C(15)-H(15B)	0.9900
C(16)-C(17)	1.5167(15)
C(17)-C(18)	1.5193(15)
C(17)-H(17A)	0.9900

C(17)-H(17B)	0.9900
C(18)-C(19)	1.4971(15)
C(18)-H(18A)	0.9900
C(18)-H(18B)	0.9900
C(16)-N(1)-C(1)	122.89(9)
C(16)-N(1)-C(15)	116.55(9)
C(1)-N(1)-C(15)	120.17(8)
C(19)-O(3)-H(3A)	108.0(11)
C(2)-C(1)-C(6)	119.49(10)
C(2)-C(1)-N(1)	120.98(10)
C(6)-C(1)-N(1)	119.53(9)
C(1)-C(2)-C(3)	120.41(11)
C(1)-C(2)-H(2)	119.8
C(3)-C(2)-H(2)	119.8
C(4)-C(3)-C(2)	120.25(11)
C(4)-C(3)-H(3)	119.9
C(2)-C(3)-H(3)	119.9
C(3)-C(4)-C(5)	120.05(11)
C(3)-C(4)-H(4)	120.0
C(5)-C(4)-H(4)	120.0
C(4)-C(5)-C(6)	120.32(11)
C(4)-C(5)-H(5)	119.8
C(6)-C(5)-H(5)	119.8
C(5)-C(6)-C(1)	119.43(10)
C(5)-C(6)-C(7)	126.74(10)
C(1)-C(6)-C(7)	113.83(9)
C(8)-C(7)-C(6)	152.26(11)
C(7)-C(8)-C(9)	154.16(11)
C(10)-C(9)-C(14)	120.70(10)
C(10)-C(9)-C(8)	126.04(10)
C(14)-C(9)-C(8)	113.25(10)
C(11)-C(10)-C(9)	119.72(11)
C(11)-C(10)-H(10)	120.1
C(9)-C(10)-H(10)	120.1
C(12)-C(11)-C(10)	120.07(11)
C(12)-C(11)-H(11)	120.0
C(10)-C(11)-H(11)	120.0

C(11)-C(12)-C(13)	120.65(11)
C(11)-C(12)-H(12)	119.7
C(13)-C(12)-H(12)	119.7
C(14)-C(13)-C(12)	120.66(11)
C(14)-C(13)-H(13)	119.7
C(12)-C(13)-H(13)	119.7
C(13)-C(14)-C(9)	118.20(10)
C(13)-C(14)-C(15)	121.10(10)
C(9)-C(14)-C(15)	120.58(10)
N(1)-C(15)-C(14)	115.63(9)
N(1)-C(15)-H(15A)	108.4
C(14)-C(15)-H(15A)	108.4
N(1)-C(15)-H(15B)	108.4
C(14)-C(15)-H(15B)	108.4
H(15A)-C(15)-H(15B)	107.4
O(1)-C(16)-N(1)	120.56(10)
O(1)-C(16)-C(17)	121.17(9)
N(1)-C(16)-C(17)	118.24(9)
C(16)-C(17)-C(18)	110.88(9)
C(16)-C(17)-H(17A)	109.5
C(18)-C(17)-H(17A)	109.5
C(16)-C(17)-H(17B)	109.5
C(18)-C(17)-H(17B)	109.5
H(17A)-C(17)-H(17B)	108.1
C(19)-C(18)-C(17)	112.12(9)
C(19)-C(18)-H(18A)	109.2
C(17)-C(18)-H(18A)	109.2
C(19)-C(18)-H(18B)	109.2
C(17)-C(18)-H(18B)	109.2
H(18A)-C(18)-H(18B)	107.9
O(2)-C(19)-O(3)	123.09(11)
O(2)-C(19)-C(18)	123.26(10)
O(3)-C(19)-C(18)	113.65(10)

Symmetry transformations used to generate equivalent atoms:

Table S15. Anisotropic displacement parameters ($Å^2 \times 10^3$) for DBCO-acid **10**. The anisotropic displacement factor exponent takes the form: $-2\pi^2[h^2a^{*2}U^{11} + ... + 2 h k a^* b^* U^{12}]$

	U^{11}	U^{22}	U^{33}	U^{23}	U^{13}	U^{12}
N(1)	20(1)	18(1)	16(1)	1(1)	0(1)	1(1)
O(1)	26(1)	24(1)	22(1)	-3(1)	4(1)	4(1)
O(2)	34(1)	19(1)	38(1)	4(1)	-14(1)	-3(1)
O(3)	28(1)	25(1)	26(1)	-7(1)	-6(1)	3(1)
C(1)	20(1)	16(1)	19(1)	-2(1)	-2(1)	-1(1)
C(2)	34(1)	22(1)	24(1)	3(1)	5(1)	-2(1)
C(3)	40(1)	17(1)	32(1)	4(1)	1(1)	-1(1)
C(4)	35(1)	17(1)	34(1)	-3(1)	1(1)	4(1)
C(5)	31(1)	21(1)	26(1)	-3(1)	5(1)	3(1)
C(6)	19(1)	17(1)	20(1)	-1(1)	-1(1)	-1(1)
C(7)	19(1)	21(1)	20(1)	0(1)	4(1)	0(1)
C(8)	19(1)	22(1)	22(1)	3(1)	5(1)	-1(1)
C(9)	18(1)	19(1)	21(1)	0(1)	-1(1)	2(1)
C(10)	24(1)	22(1)	28(1)	4(1)	5(1)	2(1)
C(11)	31(1)	18(1)	29(1)	3(1)	2(1)	4(1)
C(12)	33(1)	24(1)	30(1)	0(1)	6(1)	10(1)
C(13)	24(1)	28(1)	25(1)	2(1)	6(1)	6(1)
C(14)	18(1)	21(1)	17(1)	0(1)	-3(1)	1(1)
C(15)	17(1)	21(1)	19(1)	1(1)	0(1)	0(1)
C(16)	21(1)	17(1)	17(1)	2(1)	4(1)	-2(1)
C(17)	25(1)	21(1)	19(1)	0(1)	-1(1)	2(1)
C(18)	24(1)	22(1)	22(1)	-1(1)	-1(1)	2(1)
C(19)	23(1)	21(1)	19(1)	1(1)	2(1)	-3(1)

Table S16. Hydrogen coordinates (x 10^4) and isotropic displacement parameters ($Å^2$ x 10^3) for DBCO-acid **10**.

	x	y	z	U(eq)
H(3A)	9650(40)	5729(11)	5077(11)	57(5)
H(2)	883	3490	2692	32
H(3)	2869	2310	2310	36
H(4)	4839	2318	1162	35
H(5)	4843	3509	392	31
H(10)	2176	7317	161	30
H(11)	-445	8353	509	32
H(12)	-3467	8057	1323	35
H(13)	-3850	6734	1825	31
H(15A)	-2879	5328	1870	23
H(15B)	-1879	4990	1084	23
H(17A)	4842	4777	2917	26
H(17B)	3276	4552	3629	26
H(18A)	5510	6119	3419	28
H(18B)	3794	5941	4102	28

Table S17. Torsion angles [°] for DBCO-acid **10**.

C(16)-N(1)-C(1)-C(2)	57.57(15)
C(15)-N(1)-C(1)-C(2)	-115.02(12)
C(16)-N(1)-C(1)-C(6)	-121.80(11)
C(15)-N(1)-C(1)-C(6)	65.60(13)
C(6)-C(1)-C(2)-C(3)	2.29(18)
N(1)-C(1)-C(2)-C(3)	-177.09(11)
C(1)-C(2)-C(3)-C(4)	-1.92(19)
C(2)-C(3)-C(4)-C(5)	0.02(19)
C(3)-C(4)-C(5)-C(6)	1.48(19)
C(4)-C(5)-C(6)-C(1)	-1.09(17)
C(4)-C(5)-C(6)-C(7)	-179.93(11)
C(2)-C(1)-C(6)-C(5)	-0.79(16)
N(1)-C(1)-C(6)-C(5)	178.60(10)
C(2)-C(1)-C(6)-C(7)	178.20(10)
N(1)-C(1)-C(6)-C(7)	-2.42(14)
C(5)-C(6)-C(7)-C(8)	177.2(2)
C(1)-C(6)-C(7)-C(8)	-1.7(3)
C(6)-C(7)-C(8)-C(9)	-17.0(5)
C(7)-C(8)-C(9)-C(10)	176.5(2)
C(7)-C(8)-C(9)-C(14)	-2.3(3)
C(14)-C(9)-C(10)-C(11)	-0.58(17)
C(8)-C(9)-C(10)-C(11)	-179.28(11)
C(9)-C(10)-C(11)-C(12)	-0.07(18)
C(10)-C(11)-C(12)-C(13)	0.81(19)
C(11)-C(12)-C(13)-C(14)	-0.91(19)
C(12)-C(13)-C(14)-C(9)	0.26(17)
C(12)-C(13)-C(14)-C(15)	-175.82(11)
C(10)-C(9)-C(14)-C(13)	0.48(16)
C(8)-C(9)-C(14)-C(13)	179.34(10)
C(10)-C(9)-C(14)-C(15)	176.58(10)
C(8)-C(9)-C(14)-C(15)	-4.56(14)
C(16)-N(1)-C(15)-C(14)	65.36(12)
C(1)-N(1)-C(15)-C(14)	-121.59(11)
C(13)-C(14)-C(15)-N(1)	-121.25(11)
C(9)-C(14)-C(15)-N(1)	62.76(13)
C(1)-N(1)-C(16)-O(1)	-169.33(10)

C(15)-N(1)-C(16)-O(1)	3.51(15)
C(1)-N(1)-C(16)-C(17)	12.64(15)
C(15)-N(1)-C(16)-C(17)	-174.51(9)
O(1)-C(16)-C(17)-C(18)	-30.65(14)
N(1)-C(16)-C(17)-C(18)	147.36(10)
C(16)-C(17)-C(18)-C(19)	175.76(9)
C(17)-C(18)-C(19)-O(2)	1.16(16)
C(17)-C(18)-C(19)-O(3)	-178.03(9)

Symmetry transformations used to generate equivalent atoms:

Table S18. Hydrogen bonds for DBCO-acid **10** [Å and °].

D-H...A	d(D-H)	d(H...A)	d(D...A)	<(DHA)
O(3)-H(3A)...O(2)#1	0.93(2)	1.71(2)	2.6454(12)	177.4(18)

Symmetry transformations used to generate equivalent atoms:

#1 -x+2,-y+1,-z+1

Appendix H: *k'* data for k_2 plots

H.1. *k'* data for k_2 plot *endo*-sTCO-CH_2OH **2**

10 eq *endo*-sTCO-OH

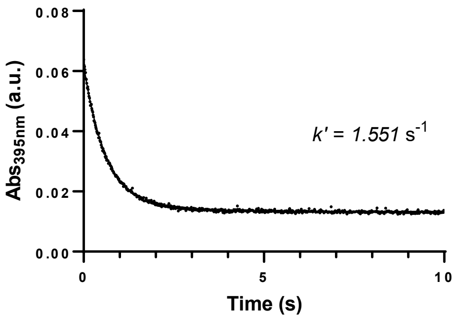

50 eq *endo*-sTCO-OH

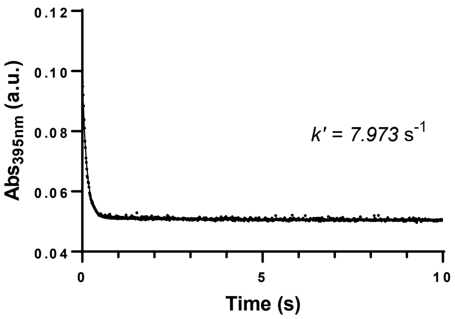

25 eq *endo*-sTCO-OH

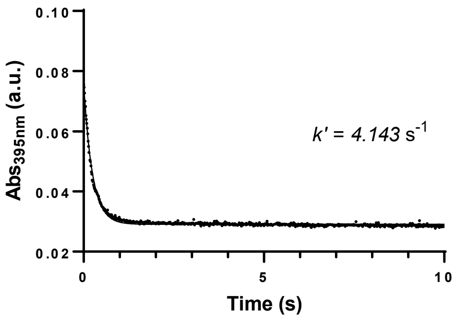

75 eq *endo*-sTCO-OH

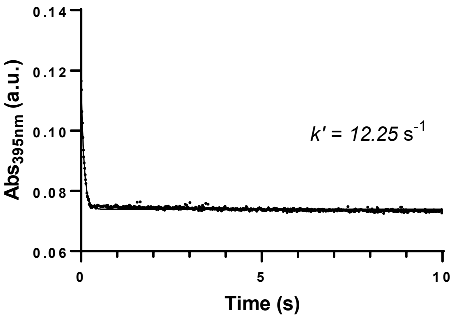

H.2. *k'* data for k_2 plot *exo*-sTCO-CH_2OH **3**

10 eq *exo*-sTCO-OH

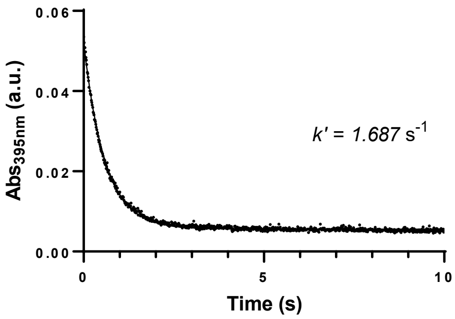

50 eq *exo*-sTCO-OH

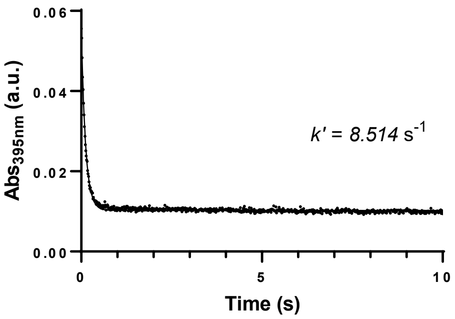

25 eq *exo*-sTCO-OH

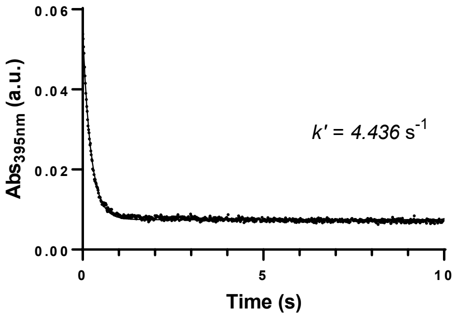

75 eq *exo*-sTCO-OH

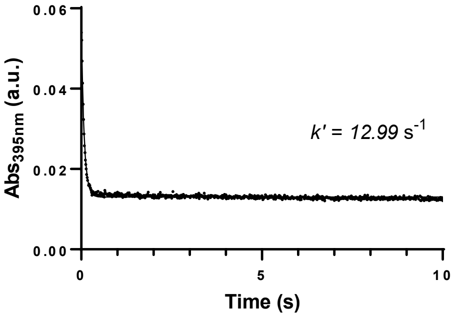

H.3. *k′* data for k_2 plot *endo*-sTCO-C(O)OEt **4**

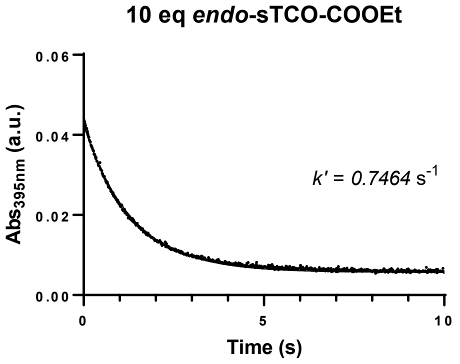

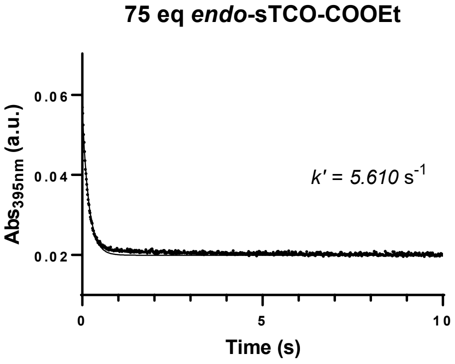

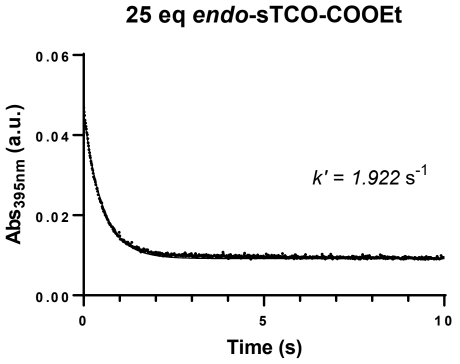

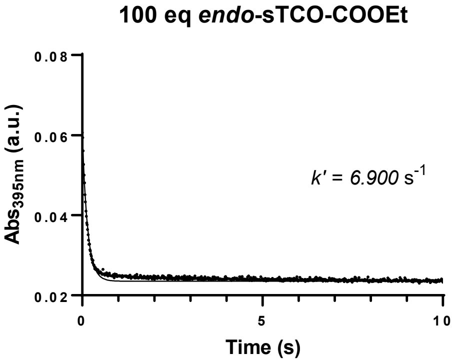

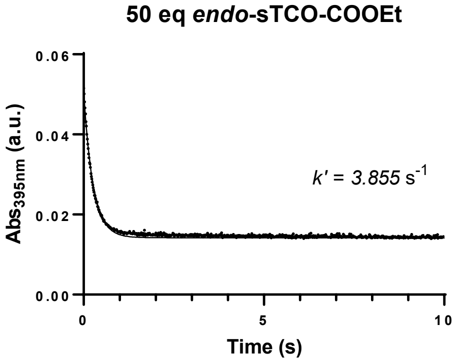

H.4. *k′* data for k_2 plot *exo*-sTCO-C(O)OEt **5**

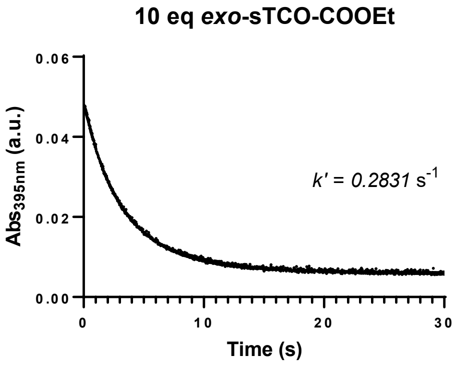

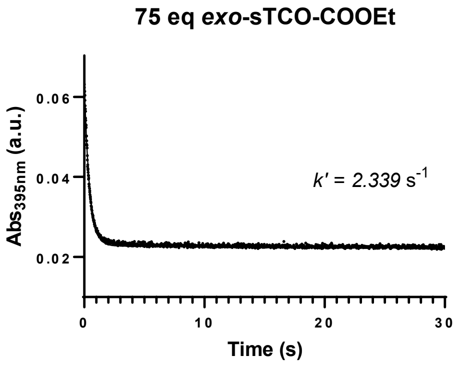

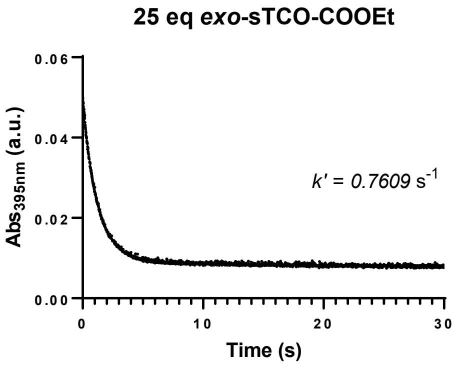

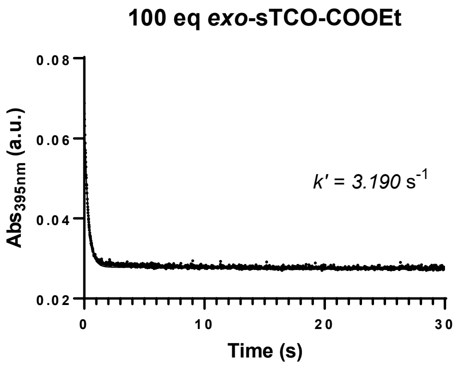

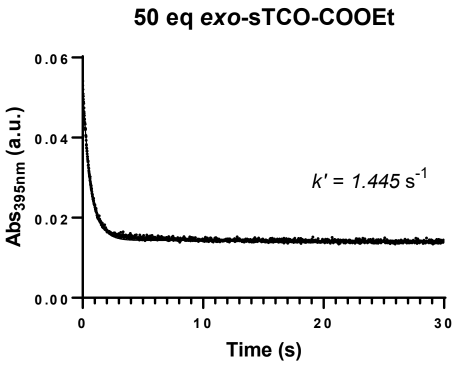

H.5. *k′* data for k_2 plot *exo*-BCN-CH_2OH **8**

10 eq *exo*-BCN-OH

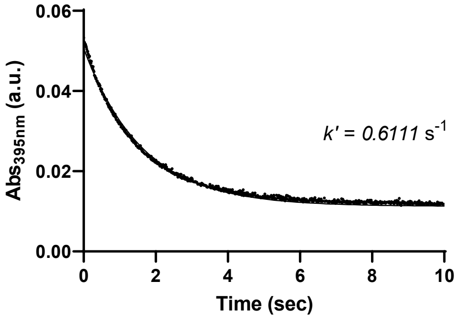

75 eq *exo*-BCN-OH

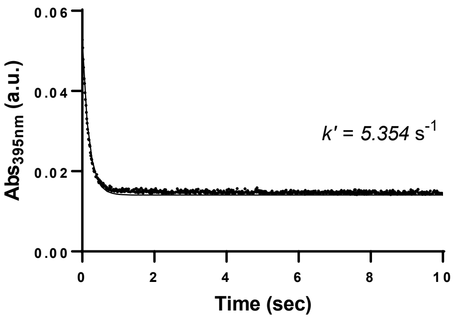

25 eq *exo*-BCN-OH

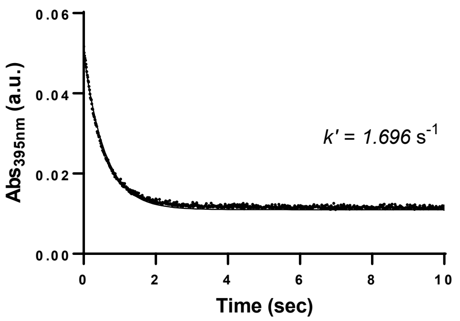

100 eq *exo*-BCN-OH

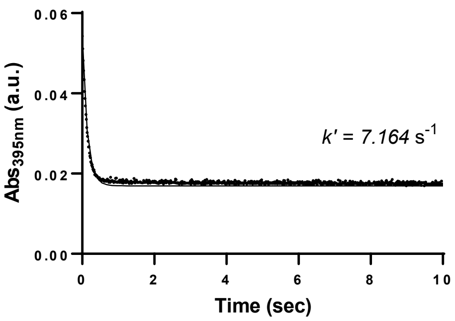

50 eq *exo*-BCN-OH

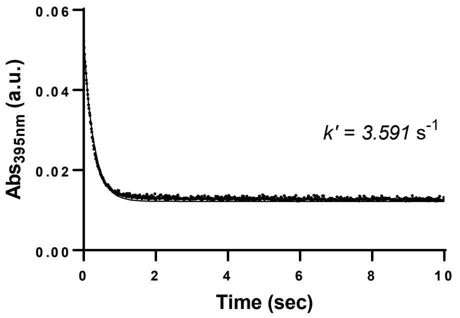

H.6. *k′* data for k_2 determination DBCO-acid **10**

10 eq DBCO-acid

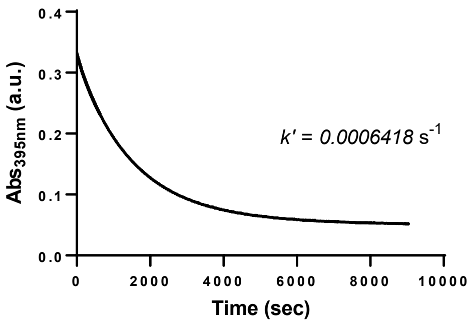

Appendix I: *k'* data of Eyring plots

I.1. *k'* data for Eyring plot *endo*-sTCO-CH_2OH **2**

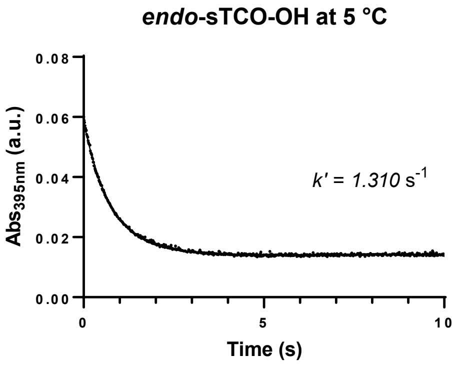

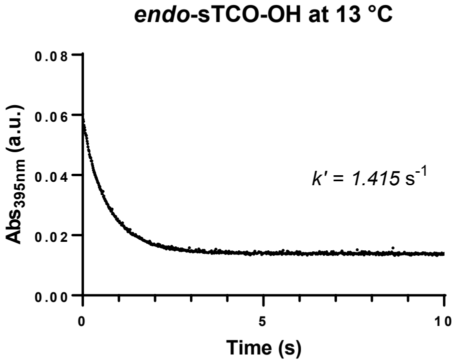

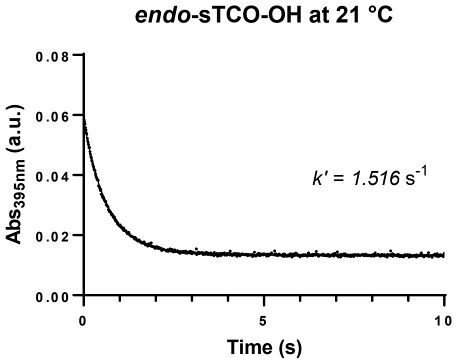

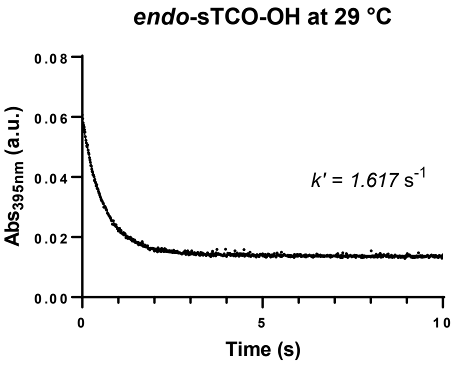

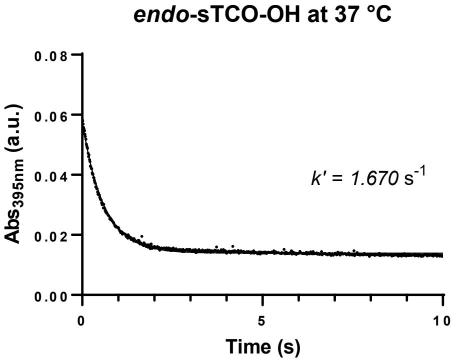

I.2. *k′* data for Eyring plot *exo*-sTCO-CH_2OH **3**

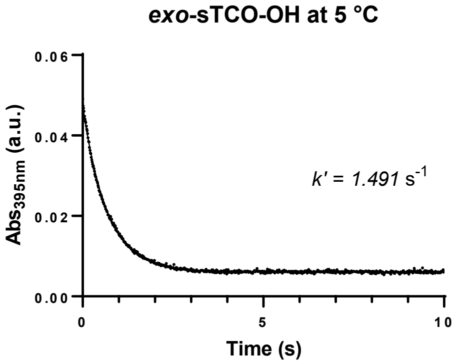

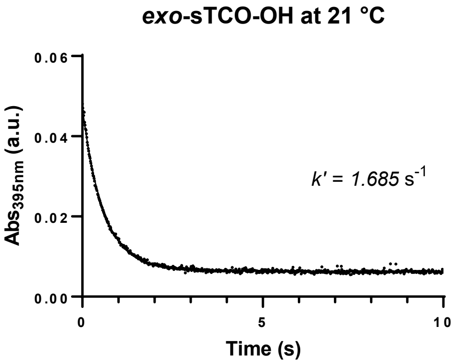

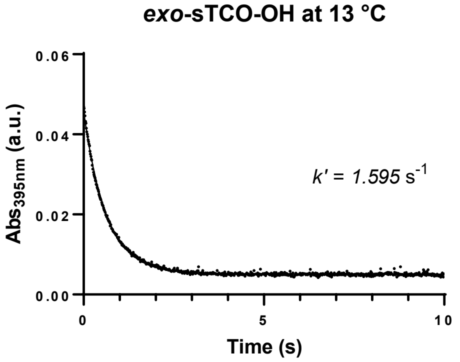

I.3. *k′* data for Eyring plot TCO-OH **6**

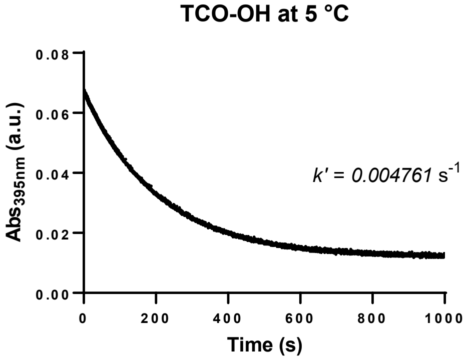

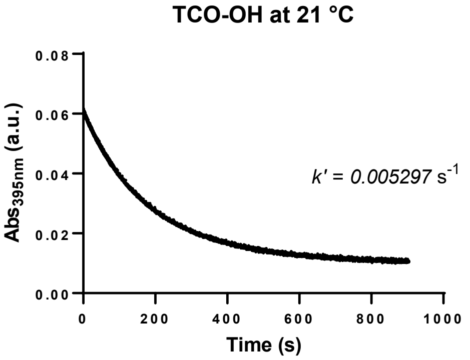

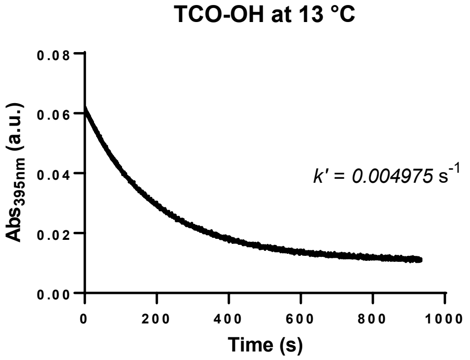

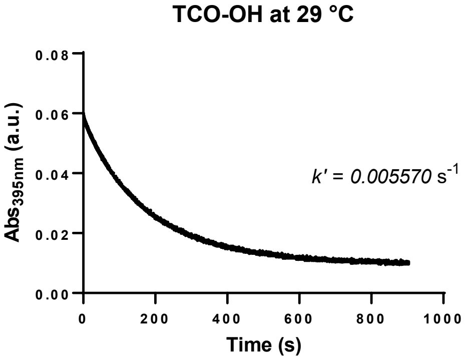

I.4. *k′* data for Eyring plot *endo*-sTCO-C(O)OEt **4**

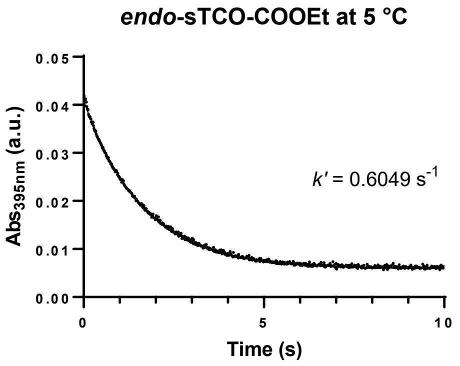

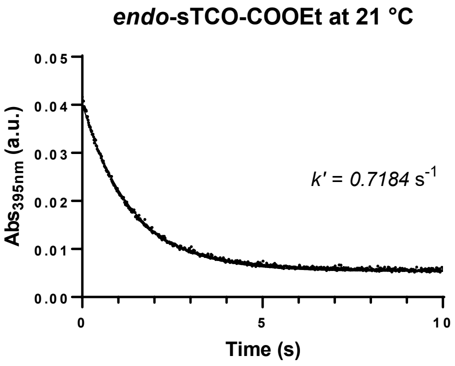

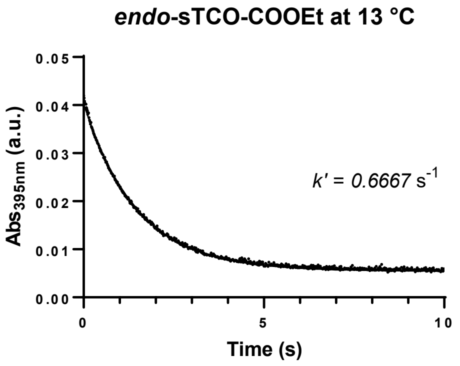

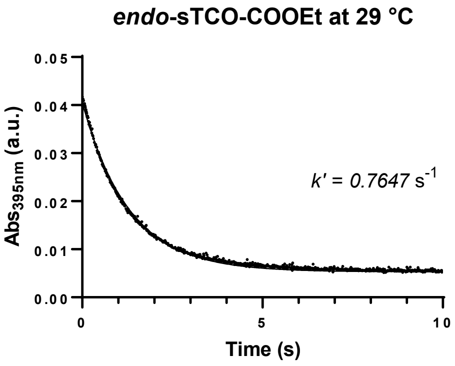

I.5. *k′* data for Eyring plot *exo*-sTCO-C(O)OEt **5**

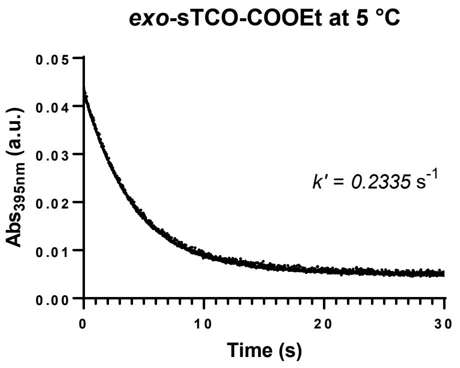

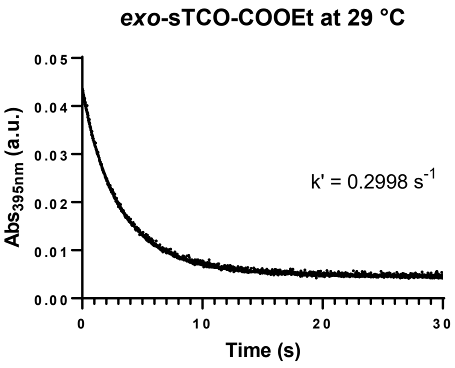

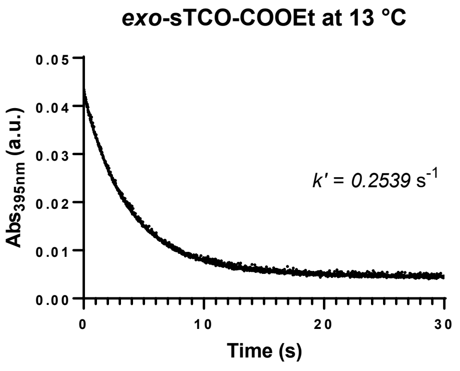

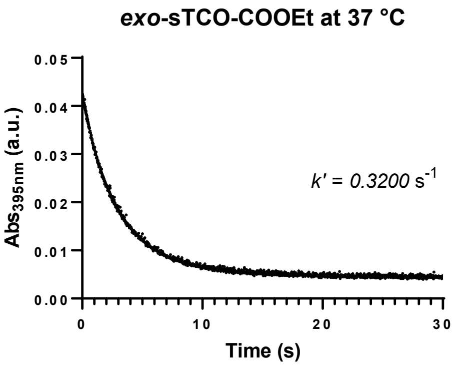

***exo*-sTCO-COOEt at 21 °C**

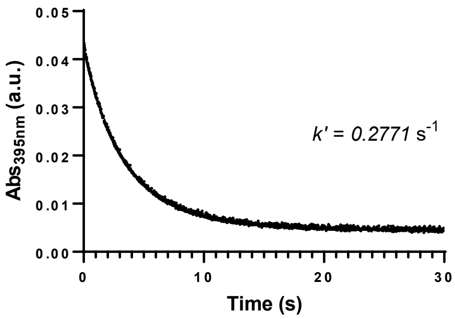

I.6. *k'* data for Eyring plot *exo*-BCN-CH_2OH **8**

***exo*-BCN-OH at 5 °C**

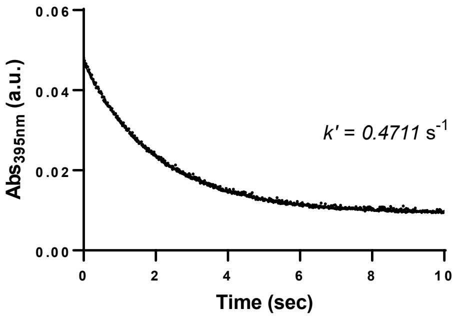

***exo*-BCN-OH at 29 °C**

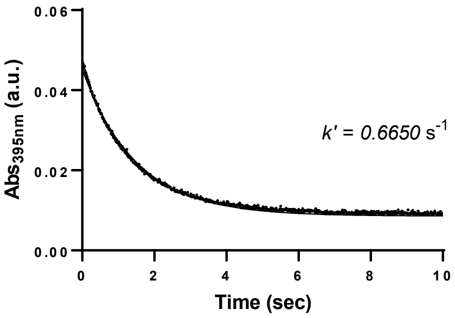

***exo*-BCN-OH at 13 °C**

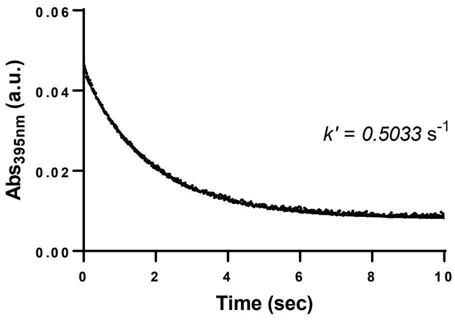

***exo*-BCN-OH at 37 °C**

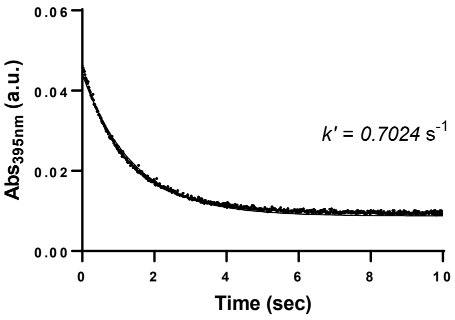

***exo*-BCN-OH at 21 °C**

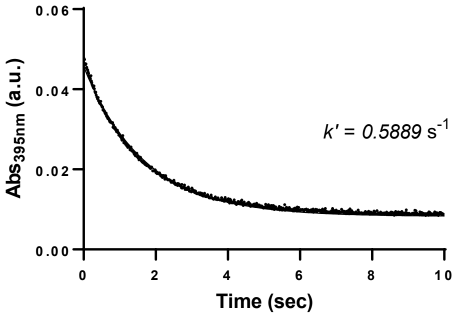

Appendix J: *k'* data of SPAAC fluorescence spectroscopy

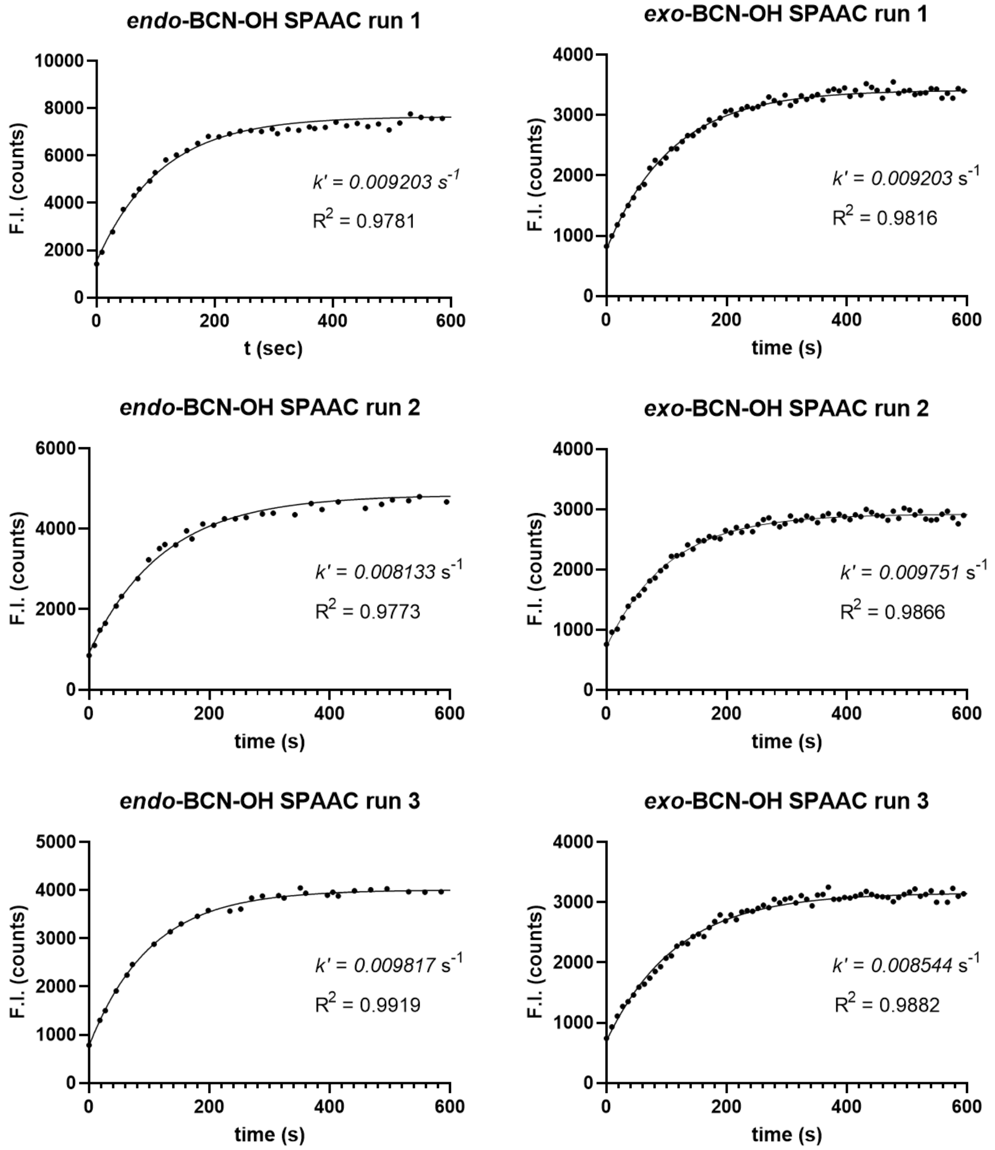

Appendix K: Heats of hydrogenation dienophiles.

Table S19. Calculated heats of hydrogenation for the dienophiles, computed at M06-2X/6-311+G(d,p) level of theory.

**Compound**	**ΔH_{hyd} (kcal/mol)**	**ΔG_{hyd} (kcal/mol)**
endo-sTCO-CH_2OH (**2**)	−24.8	−25.6
exo-sTCO-CH_2OH (**3**)	−25.2	−25.9
endo-sTCO-C(O)OEt (**4**)	−28.0	−28.3
exo-sTCO-C(O)OEt (**5**)	−27.1	−27.6
TCO-OH (**6**)	−25.4	−26.7
endo-BCN-CH_2OH (**7**)	−23.4	−24.0
exo-BCN-CH_2OH (**8**)	−21.2	−24.0
THS (**9**)	−27.6	−28.2

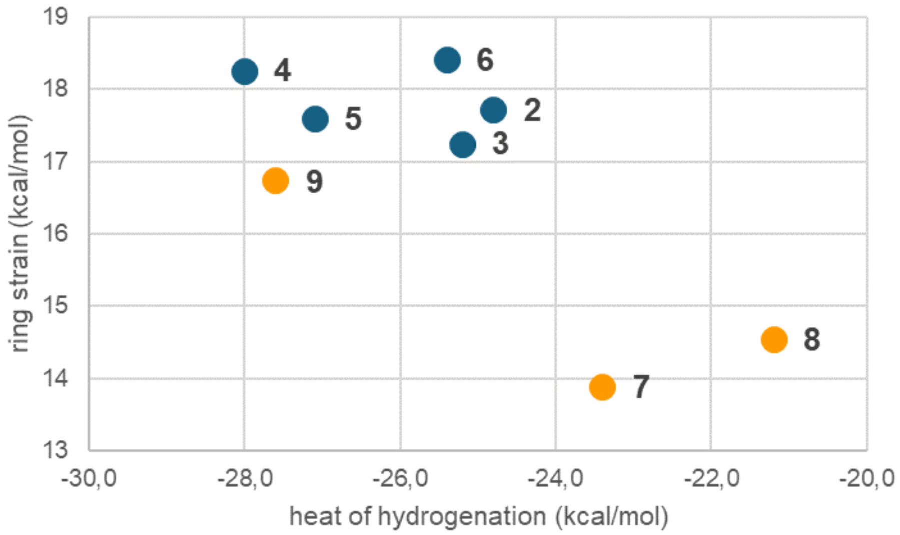

Figure S3. Scatter plot of the calculated ring strain (kcal/mol) versus heat of hydrogenation (kcal/mol) for the dienophiles used in this study (blue: alkenes, orange: alkynes).

Appendix L: Calculated balance of the entropies and enthalpies of activation.

Table S20. Thermodynamic parameters were computed by means of DFT calculations with the Gaussian 16 suite of computational programs. The geometries of all stationary points were optimized using the Minnesota family of density functional M06-2X and the 6-311+G(d,p) basis set. The solvent effect of a mixture of water:MeOH (ε=56.28) was evaluated implicitly by using the SMD continuum solvation model.

**Compound**	**Δ*H*‡ (kcal/mol)**	**TΔ*S*‡ (kcal/mol)**	**Δ*G*‡ (kcal/mol)**
endo-sTCO-CH_2OH (**2**)	–0.7	12.6	13.4
exo-sTCO-CH_2OH (**3**)	–0.7	12.5	13.2
endo-sTCO-C(O)OEt (**4**)	–0.6	13.0	13.6
exo-sTCO-C(O)OEt (**5**)	–0.4	13.3	13.7
TCO-OH (**6**)	2.8	19.7	17.0
endo-BCN-CH_2OH (**7**)	1.5	16.3	14.8
exo-BCN-CH_2OH (**8**)	1.3	16.2	15.0
THS (**9**)	0.5	16.5	16.0
